# Implementation barriers and facilitators to a COVID-19 intervention in Bangladesh: The benefits of engaging the community for the delivery of the programme

**DOI:** 10.1186/s12913-022-08939-7

**Published:** 2022-12-28

**Authors:** Fahmida Akter, Malika Tamim, Avijit Saha, Imran Ahmed Chowdhury, Omor Faruque, Animesh Talukder, Mohiuddin Ahsanul Kabir Chowdhury, Monzur Morshed Patwary, Albaab-Ur Rahman, Morseda Chowdhury, Malabika Sarker

**Affiliations:** 1 BRAC James P Grant School of Public Health, BRAC University, Dhaka, Bangladesh; 2 Health, Nutrition, and Population Program, BRAC, Dhaka, Bangladesh; 3 Heidelberg Institute of Global Health, Heidelberg University, Heidelberg, Germany

**Keywords:** COVID-19, Community resilience, Community engagement, Implementation fidelity, Facilitators, And Barriers

## Abstract

**Background:**

BRAC (Bangladesh Rural Advancement Committee), the largest NGO globally, implemented a community-based comprehensive social behavior communication intervention to increase community resilience through prevention, protection, and care for COVID-19. We conducted implementation research to assess fidelity and explore the barriers and facilitators of this intervention implementation.

**Methods:**

We adopted a concurrent mixed-method triangulation design. We interviewed 666 members of 60 Community Corona Protection Committees (CCPCs) and 80 members of 60 Community Support Teams (CSTs) through multi-stage cluster sampling using a structured questionnaire. The qualitative components relied on 54 key informant interviews with BRAC implementers and government providers.

**Results:**

The knowledge about wearing mask, keeping social distance, washing hands and COVID-19 symptoms were high (on average more than 70%) among CCPC and CST members. While 422 (63.4%) CCPC members reported they ‘always’ wear a mask while going out, 69 (86.3%) CST members reported the same practice. Only 247 (37.1%) CCPC members distributed masks, and 229 (34.4%) donated soap to the underprivileged population during the last two weeks preceding the survey. The key facilitators included influential community members in the CCPC, greater acceptability of the front-line health workers, free-of-cost materials, and telemedicine services. The important barriers identified were insufficient training, irregular participation of the CCPC members, favouritism of CCPC members in distributing essential COVID-19 preventive materials, disruption in supply and shortage of the COVID-19 preventative materials, improper use of handwashing station, the non-compliant attitude of the community people, challenges to ensure home quarantine, challenges regarding telemedicine with network interruptions, lack of coordination among stakeholders, the short duration of the project.

**Conclusions:**

Engaging the community in combination with health services through a Government-NGO partnership is a sustainable strategy for implementing the COVID-19 prevention program. Engaging the community should be promoted as an integral component of any public health intervention for sustainability. Engagement structures should incorporate a systems perspective to facilitate the relationships, ensure the quality of the delivery program, and be mindful of the heterogeneity of different community members concerning capacity building. Finally, reaching out to the underprivileged through community engagement is also an effective mechanism to progress through universal health coverage.

**Supplementary Information:**

The online version contains supplementary material available at 10.1186/s12913-022-08939-7.

## Background

The first case of COVID-19 patient was detected in Bangladesh on March 8, 2020. Since then, a total of 1.94 million people have been officially reported as COVID-19 patients, with the death toll now over 29,000 [1]. Although the Government of Bangladesh enforced national-level lockdowns to contain the spread of the virus, the country’s biggest obstacles were inadequate healthcare service facilities and the lack of public awareness to undertake preventive health measures [2].

The World Health Organization (WHO) advises using masks, maintaining hand hygiene, keeping physical distance of at least 1 m and avoiding touching one’s face as a comprehensive package of prevention and control measures to limit the spread of COVID-19. Other infection prevention and control (IPC) measures include adequate ventilation in indoor settings, testing, contact tracing, quarantine, and isolation. Together these measures are critical to prevent human-to-human transmission of SARS-CoV-2 [3].

Previous studies have notably found that following protocols such as mask-wearing, handwashing, and maintaining social distance reduce the transmission of infection and, thus, fatalities [4–6]. According to recent studies, preventive measures and proper hygiene etiquette are needed even after vaccination to substantially limit the transmission of the virus if the vaccination coverage is not optimal [7–9]. However, Bangladesh has faced significant structural barriers in combating COVID-19 with conventional restrictive methods due to its high population density, many living on a daily wage, high rate of poverty, and an overall weak healthcare system. Even though COVID-19 immunization has begun in many countries, widespread vaccination campaigns in a short period remain a challenge in Bangladesh [10]. Thus, there is an immediate need for extensive behavioural change initiatives via knowledge dissemination to ensure understanding and compliance with the COVID-19 preventative protocols at the community level [9, 11].

The local actors (local authorities, religious leaders, NGOs, and youth and women groups) in every community are critical to generating demand and negotiating for high-quality, people-centered health care and strengthening the health system due to their intersecting relationships with the health sector [3, 12]. The community health workforce may effectively boost the primary healthcare related to the COVID-19 response as they are trusted in the community and hold an important relationship with the facilities, leaders, and organizations. Moreover, a community-based approach can decrease the fear and stigma of the disease and enhance community participation and build healthcare capacity, and thus, can create resilience for the re-emergence of any lethal illness in the future, reducing the need for harsh measures such as lockdowns [12]. BRAC, the largest NGO in the world, has emerged as one of the most vital community-level implementation partners in Bangladesh’s overall COVID-19 response. BRAC Health, Nutrition, and Population Programme (HNPP) implemented the ‘Comprehensive COVID-19 Response through Community Mobilization and Strengthening Community Clinics’ project to ensure COVID-19 prevention, protection, and care through community engagement and participation. BRAC recognizes that creating community resilience to prevent COVID-19 requires the involvement of the local communities.

Despite a growing body of evidence on COVID-19 mitigation approaches, to date, to our knowledge, no research has been done to identify the challenges or the enabling factors of a community-based COVID-19 mitigation project in the rural areas of Bangladesh. In particular, engaging key community members for COVID-19 mitigation is also a unique approach. If successful, this type of initiative can be scaled up. Our study aimed to assess the implementation fidelity of BRAC’s COVID-19 project and explore the barriers and facilitators of this COVID-19 project while creating community resilience for COVID-19 responses. Thus, the findings from this study can inform designing and planning of future COVID-19 mitigation projects adopting a novel approach like community engagement in combination with health service through Government-NGO partnerships in similar contexts.

## Methods

### Description of the intervention

**Fig. 1:**
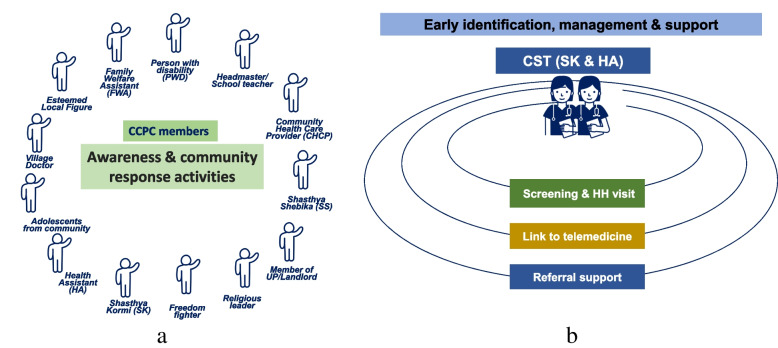
**a** Composition of Community Coronavirus Protection Committee (CCPC). **b** Composition of Community Support Team (CST) with roles and responsibilities

As one of the interventions, BRAC created the “Community Corona Protection Committee” (CCPC), each comprising approximately 15 members drawn from community groups, community support groups, government, BRAC, and other community representatives at the community clinic (CC) level (Fig. 1-a). BRAC recruited them as volunteers and trained them about COVID-19 symptoms, transmission, prevention, and protection, followed by monthly refresher training. The CCPCs acted as social and behavioral change communication (SBCC) agents and educated the community on COVID-19 preventive measures. For primary prevention, they distributed free-of-cost COVID-19 safety gear i.e., masks and handwashing supplies and materials in their community.

**Fig. 2:**
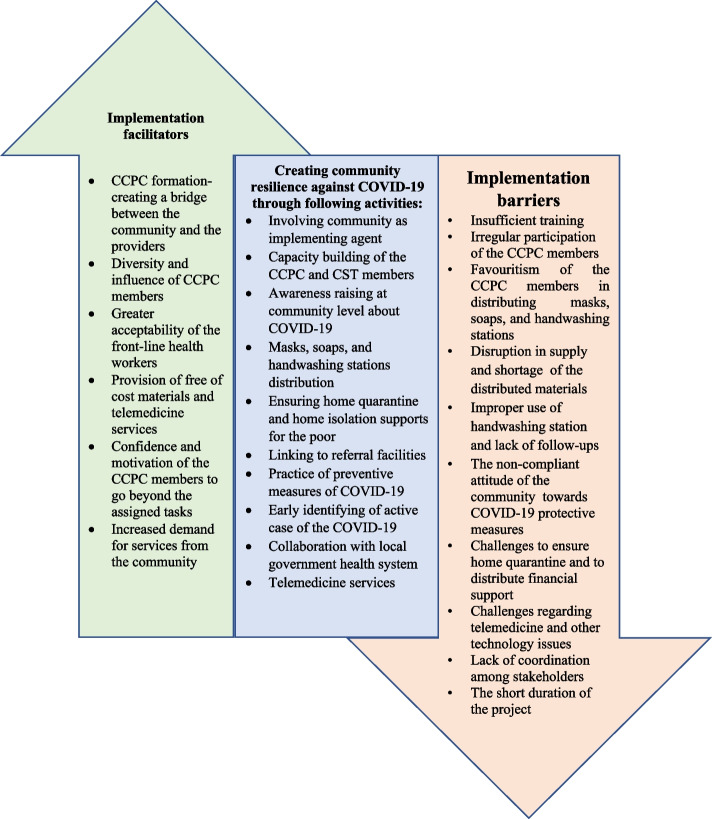
Implementation facilitators and barriers while creating community resilience against COVID-19 pandemic situation

Moreover, two-member (*Shasthya Kormi*/SK and Health assistant/HA) community support teams (CSTs) were created at the union level to assist the community with symptom diagnosis, telemedicine services, and healthcare referrals (Fig. 1-b) to promote secondary prevention. Supplementary Table 1 and Fig. 2 show the project activities intended to create community resilience from adaptive perspectives [13, 14]. However, several activities could also contribute to other aspects of resilience besides the adaptive perspective. For example, collaboration with the government at the local level and the provision of telemedicine services can help create transformative resilience in the long run. We sought to ascertain if investing in a community-based intervention would enable implementers to better adapt to adversity and, in turn, benefit their community via increased capability.

### Research design and sites

**Fig. 3:**
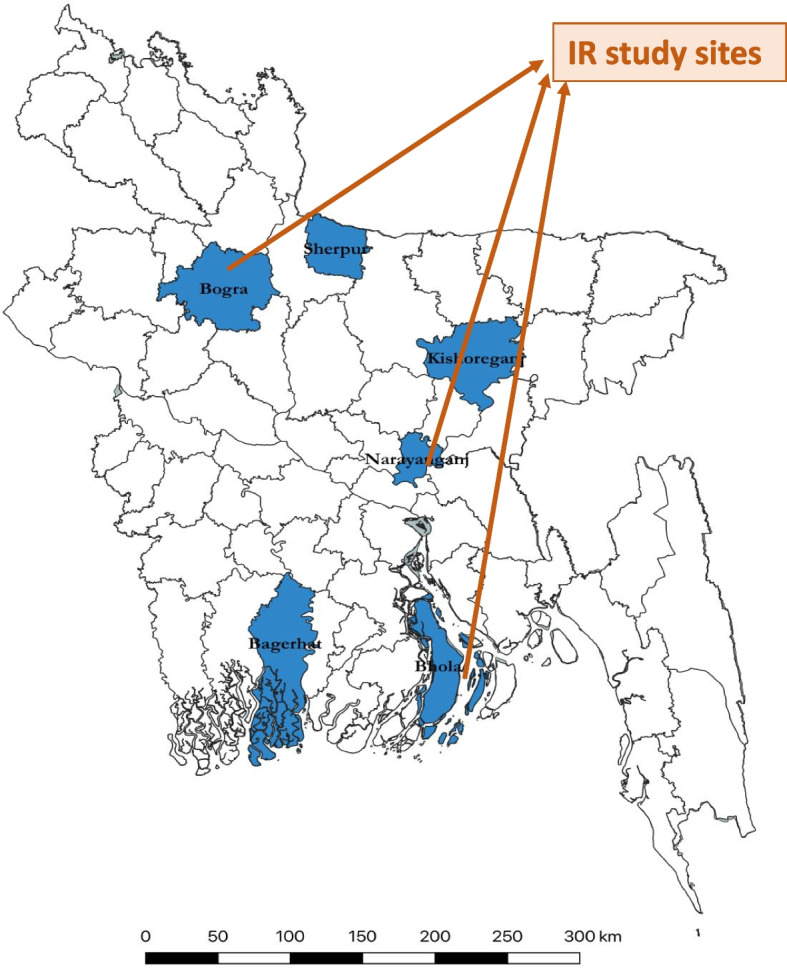
Six intervention districts (in blue color) and Implementation Research (IR) study sites (three districts: Bhola, Bogura, and Narayanganj) indicated by orange-colored arrows (source: authors)

This study used a concurrent mixed-method triangulation methodology to analyse implementation fidelity, barriers, and best practices of the COVID-19 project during the intervention stage. We used the Standards for Reporting Implementation Studies (StaRI) Statement as a checklist for standard reporting (see Supplementary File 2) [15]. A survey was carried out to assess adherence, and a qualitative approach was employed to identify barriers and best practices. Out of 20 ‘Better Health Bangladesh' districts, six were chosen based on high COVID-19 infection rates, low service usage rates (4 Antenatal Care/ANC compliance among pregnant women), and operational viability as BRAC’s intervention districts. We conducted our survey between March 23 and April 8, 2021, in three (Bhola, Bogura, and Narayanganj) of the project’s six districts which represented coastal region, highlands, and plain land, respectively (Fig. 3), and were selected because of the high COVID-19 caseload. From each of those districts, three sub-districts were selected as study sites using simple random sampling.

### Study population, sampling strategy and sample size

We collected qualitative and quantitative data from randomly selected three sub-district volunteers, CCPC and CST members, implementers from BRAC at different levels, and government healthcare providers using a multi-stage cluster sampling.

### Quantitative

The quantitative components included a survey and a direct observation using a checklist. The survey involved CCPC and CST members from the research locations. A list of all CCs (as CCPCs are CC-centric) within the designated sub-districts was collected from BRAC HNPP. Finally, we randomly chose 20 CCPCs and 20 CSTs from each sub-district and interviewed all CCPC and CST members (Supplementary Table 2). In total, 666 CCPC members participated in our study, out of whom 30% were from Bhola, 38% from Bogura, and 30% from Narayangonj. Table 1 illustrates the CCPC & CST members' distribution by category. In addition, we interviewed 80 CST members. Besides, ninety (equally distributed in three districts) handwashing stations were conveniently selected for direct observation.

**Table 1:** Profile of CCPC and CST members

Member's distribution	n (%)
*CCPC*	*Total (N* = *666)*
Community	
Esteemed local figure	193 (28.98)
Other community representative	160 (24.02)
Headmaster/schoolteacher	47 (7.06)
Adolescent girl/boy	42 (6.31)
Freedom fighter	14 (2.1)
Religious leader	13 (1.95)
Village doctor	11 (1.65)
Person with disability	7 (1.05)
Governmental	
Union Parishad member/land donor (Jomidata)	70 (10.51)
Community health care provider (CHCP)	53 (7.96)
Family welfare assistant (FWA)	30 (4.5)
Health assistant (HA)	12 (1.8)
BRAC	
*Shasthya Shebika* (SS)	9 (1.35)
*Shasthya Kormi* (SK)	5 (0.75)
*CST*	*Total (N* = *80)*
*Shasthya Kormi* (SK)	40 (50.00)
Health assistant (HA)	39 (48.75)
Family welfare assistant (FWA)	1 (1.25)

### Qualitative

We performed Key Informant Interviews (KIIs) with BRAC implementers to identify obstacles and best practices in conducting comprehensive COVID-19 response operations (Supplementary Table 3). The non-CCPC-CST interviewees were field organizers (FOs), telemedicine doctors, area managers (AMs), and divisional managers at BRAC. The purposive sampling technique was used to obtain extensive and rich information about their involvement with the BRAC COVID-19 project, hierarchy, geographical coverage, and gender.

### Study tools

The study tools (questionnaire and guide for KII) used in this study were developed for this study by the study investigators (see Supplementary file 1).

### Quantitative survey tools

The structured questionnaires inquired information about respondents' backgrounds, general information about the COVID-19 project and related training they received, knowledge and practices regarding COVID-19 prevention and vaccination, and roles and responsibilities of CCPC/CST members. Besides, a brief observation checklist was developed to assess the usability/functionality and current usage of the handwashing stations provided by BRAC HNPP.

### Qualitative interview guideline

Topic guide and protocol for KII were developed consisting of all the components, including barriers, facilitators, best practices, and suggestions to overcome the barriers of the project while making the resilient community for COVID-19 responses.

### Variables of interest

From the evaluation perspective, the main outcome variable was implementation fidelity which was assessed from information collected through the quantitative component. Implementation fidelity refers to how closely an intervention follows its planned implementation [16]. In this study, we focused on adherence to the intervention design and process. We collected information regarding training, knowledge, and practices relevant to COVID-19 prevention, and vaccination, from the members of CCPC and CST to assess implementation fidelity. Our other outcome variables were barriers and facilitators of the intervention, which were explored through the qualitative component.

### Data collection process

Despite the COVID-19 pandemic, we attempted to conduct all the interviews in person (excluding telemedicine doctors and BRAC headquarters employees), taking all necessary safeguards. The telemedicine doctors and BRAC headquarters personnel were interviewed by phone and Google Meet (a web-based communication service) because of COVID-19 restrictions at the time of data collection in office settings. Both quantitative and qualitative data collectors received two days of in-person training. An additional orientation on safety procedures against COVID-19 was conducted. The field supervisors monitored the data collection and conducted a daily briefing with the data collectors.

### Quantitative data collection

The survey was conducted by 18 Field Research Assistants who were divided into three teams, each led by one supervisor to collect data from three districts. The structured questionnaires were used in face-to-face survey interviews. The team worked with BRAC HNPP project officials to compile a list of interviewees (CCPC and CST members). We covered all accessible CCPC and CST members. The data was collected using Android devices in SurveyCTO version 2.70.

### Qualitative data collection

The KIIs were conducted face-to-face (except five by phone or Google Meet), and all COVID-19 pandemic-related precautions were implemented for both interviewers and responders, which lasted roughly 40 min.

### Data management and analysis

#### Quantitative

The quantitative data, after initial cleaning and quality assurance, were analyzed mainly for descriptive statistics. We checked for frequency distribution with percentage for the categorical or nominal variables and extracted mean with standard deviations for the continuous variables. All these analyses were executed using Stata version 15.

#### Qualitative

All interviews were audio-captured and then transcribed in Bangla. The qualitative analysis was performed manually using thematic analysis [17], a method for detecting, analysing, and reporting themes or patterns within data to organize and characterize the data set in detail. The data analysis was driven by the research objectives. Along with deductive coding, we examined inductive coding. The data were coded to fit into pre-existing or pre-determined themes and coding frames based on study objectives. The data in the matrix was organized using Microsoft Excel. Themes and sub theme emerged from the findings. Two authors independently read subset of the transcripts. They checked the coding scheme and the coding process to check the consistency of the emerging interpretation of the data.

Where applicable, the survey's quantitative data were triangulated with the implementers' qualitative data (methodological triangulation) to provide comprehensive information. The qualitative data corroborated various themes and supported the quantitative conclusions. Besides we also used data from the observation of handwashing stations to confirm relevant data from qualitative and quantitative components. We also performed ‘data triangulation’ as we collected information on the same topic from different levels of respondents (field level vs. headquarter level; BRAC health workers vs. government health providers etc.).

### Ethical consideration

The Institutional Review Board (IRB) of BRAC University's James P Grant School of Public Health (IRB-20-November'20–049) approved the study. All respondents signed an informed consent form after being told of the study's objectives, voluntary participation, and the freedom to withdraw at any moment during the interview. The responses were also given a unique ID to protect the respondents' privacy. We also informed them about the risks of COVID-19 transmission. Both interviewers and responders followed all safety protocols. Both interviewers and respondents wore masks before the interviews. The interviewers also sanitized their hands and kept a 3-feet distance.

## Results

### Background characteristics of the survey respondents

Among the CCPC members (N = 666), the majority were male 404 (60.7%), and the mean age was 42 years. The majority, 369 (55.4%) completed grade 10 or higher. On the other hand, 45 out of 80 CST members were female. The mean age of the CST members was 35 years, and 64 (80.0%) of them completed grade 12. Among the KII respondents, 30 out of 54 were female, with a mean age of 35.5 years. Forty-four respondents completed at least 12^th^ grade.

### Assessment of community implementors

#### Orientation and refreshers for the CCPC and CST members

More than two-thirds of 472 (70.9%) CCPC members participated in the BRAC-organized orientation/training to familiarise themselves with the COVID-19 project's operations. "General understanding of COVID-19," "Signs and symptoms, together with transmission techniques, and COVID-19 prevention activities" were the most recollected subjects from the orientation session (Supplementary Table 4). Those who missed or did not receive the orientation meetings were not notified or were busy during training. However, 262 (39.3%) of the CCPC members received refresher training. Overall, 65 (85.0%) CST members participated in an orientation session, but only 52 (65,0%) received refresher training.

### Knowledge related to COVID-19 among CCPC and CST members

Most CCPC members indicated fever/chills, cough, sore throat, shortness of breath, and headache as COVID-19 symptoms. Overall, 645 (98.02%) reported fever/chills, 616 (93.62%) reported cough, 439 (66.72%) reported sore throat, 356 (54.10%) reported shortness of breath, and 230 (34.95%) reported headache. Six-hundred and nine (93.1%) CCPC members mentioned the ‘droplets originated from coughing/sneezing’ and 463 (70.8%) mentioned ‘touching an infected human’ can transmit COVID-19. Among all CCPC members, 585 (89.2%), 472 (72.0%), and 471 (71.8%) reported ‘washing hands regularly with soap and water’, ‘maintaining social distancing’, and ‘wearing a face mask while going out’, respectively as necessary preventive measures.

The CST members echoed the CCPC members' key symptoms. Among the CST members, 80 (100.0%) reported fever/chills, 76 (95.0%) reported cough, 66 (80.5%) reported sore throat, 38 (47.5%) reported shortness of breath, and 30 (37.50%) reported congestion/runny nose. Seventy-seven (96.3%) CST members mentioned the ‘droplets originated from coughing/sneezing’ and 65 (81.3%) mentioned ‘touching an infected human’ can transmit COVID-19. The number of CST members who reported ‘washing hands regularly with soap and water’, ‘maintaining social distancing’, and ‘wearing a face mask while going out’ respectively as necessary preventive measure was 75 (93.8%), 69 (86.3%), and 64 (80.0%), respectively. Only 17 (12.5%) CST members stated that washing hands is required after blowing the nose/coughing, respectively.

### Practice related to COVID-19 among CCPC and CST members

Only 422 (63.4%) CCPC members reported they ‘always’ wear a mask while going out, which was comparatively higher for CST members 69 (86.3%).

### Tasks related to the COVID-19 project performed by the CCPC members and CST members

The CCPC and CST members were responsible for a variety of roles and tasks as per project protocol. Creating community awareness 556 (83.5%) and providing COVID-19 protection supplies 391 (58.7%) were reported as the most critical responsibilities by the majority (Table 2). Through 'individual engagement' 483 (72.5%), 'community meeting' 334 (50.3%), and 'communication with BRAC staff' 193 (29.0%), they completed their job. The main task for CST members was to ‘Identify Potential Infected Individuals/Screened Positive Suspected cases in their community’, although only 27 (67.5%) of the SK and 28 (70.0%) of the HAs mentioned it (Table 2).

**Table 2:** Performed tasks by the CCPC & CST members any time in the project duration

List of tasks	n (%)	
**Performed by CCPC members**	**Total (N = 666)**	
Creating community awareness about COVID-19	556 (83.48)	
Distributing masks and soap to poor and ultra-poor	391 (58.71)	
Arranging cost-effective handwashing stations	149 (22.37)	
Encourage people to get vaccinated	148 (22.22)	
Encourage getting tested for people with COVID-19 symptoms	146 (21.92)	
Making referral linkage to doctor/health facility	87 (13.06)	
Help identify presumptive COVID-19 cases	72 (10.81)	
Make a list of the poor and ultra-poor in the community	67 (10.06)	
Extend food and other support to people undergoing isolation	65 (9.76)	
Attend/ arrange monthly meetings	56 (8.41)	
Ensure maintenance of handwashing stations in the community clinic	37 (5.56)	
Accompany registered individuals for vaccination	33 (4.95)	
Accompany registered individuals for printing vaccination card	24 (3.60)	
Monitor/complete social audit tool checklist	16 (2.40)	
**Performed by CST members**	**(N = 80)**	
	**SKs** **n = 40**	**HAs** **n = 40**
Identify Potential Infected Individuals/ Screened Positive Suspected Case	27 (67.50)	28 (70.00)
Assess own health and take proper safety precautions	26 (65.00)	22 (55.00)
Ensure home quarantine	23 (57.50)	21 (52.50)
Connect individuals with symptoms to telemedicine	19 (47.50)	6 (15.00)
When necessary, refer to the nearest COVID-19 testing/treatment facility	15 (37.50)	10 (25.00)
Ensure home quarantine of suspected cases identified and ensure financial help for them	9 (22.50)	12 (30.00)
Identify people eligible for COVID-19 vaccinations	11 (27.50)	10 (25.00)
Do the online registration for people eligible for COVID-19 vaccinations	5 (12.50)	11 (27.50)
Keep contact with CCPC members	9 (22.50)	5 (12.50)
Enter patient data into tablets	11 (27.50)	3 (7.50)
Give health education/advice to PIIs and their family members	8 (20.00)	4 (10.00)
Ensure maintenance of handwashing station at CC	4 (10.00)	4 (10.00)
Raise awareness on three key protective measures during HH visits	1 (2.50)	2 (5.00)

In addition, we explored the specific tasks performed to increase community-based protection against COVID-19 in the last week preceding the survey. About one-third of those interviewed, distributed masks 247 (37.1%), and donated soap 229 (34.4%) to the poorest groups in the community. As of the interview date, the CCPC supplied an average of 62 masks and 36 soaps and helped six low-income families and prepared for home quarantine/isolation. Along with home isolation and quarantine assistance, they offered four types of support; 60 (46.2%) delivered medication, 77 (59.2%) helped with food delivery, 31 (23.9%) referred the patients to nearby healthcare institutions, and 67 (51.5%) helped with telemedicine counselling.

Among the CST members, 29 (36.3%) offered financial assistance (47.5% of the SK and 25.0% of the HAs) to poor/ultra-poor households on behalf of BRAC. Although 59 (73.3%) of the CST members referred COVID-19 patients for testing and 18 (22.5%) accompanied them, only 16 (20.0%) presented a referral slip. Fifty-nine (73.8%) CST members (85.0% of the SK and 62.5% of the HAs) identified the eligible candidates for vaccination ‘during their household visit.’

### Implementation facilitators

#### CCPC formation- creating a bridge between the community and the providers

The CCPC represented community representatives and community-level healthcare providers. Therefore, engaging CCPC members as implementers was a crucial strategy for the success of this project. Most CCPC members were influential community members, well-respected, and essential in raising people's awareness. The CCPC members created a bridge between the community and service providers. Their participation led to increased trust in the community about the project's activities.

*"Because of the CCPC committee, village people are aware of COVID-19. Those suffering from fever, cough and cold are informing the CCPC members, who are sending the patients to government health providers." (HA, Govt., KII 27)*

### Diversity and influence of CCPC members

The diversity in the composition of the CCPC further strengthened community resilience. As the CCPC included political leaders, religious leaders, community health workers, and adolescents, diverse perspectives were represented in the committee. Both males and females from the community were represented. Due to the diverse representation in the committee, there was less overlap of roles or competition between the CCPC members. Members could collaborate well and work on the project from their positions in society which contributed to building social cohesion.

*“Almost all CCPC members are influential; Union Parishad member, family welfare assistant (FWA), HA, Imam That is why CCPC is very effective…Every Friday, the Imam tells the people about the need for following BRAC's COVID-19 prevention activities which further enhances the acceptability of this committee.” (FO, BRAC, KII 32)*

### Greater acceptability of the front-line health workers

The acceptability of the implementers by the community facilitated the execution of the project. The community had a strong sense of trust and an optimistic view of both BRAC and Government health workers, as they were well-known and had been working door-to-door in those communities for many years. It eased the implementer’s access to the community and helped them accomplish the project objectives.

*“People know me and trust me, so they give me their cards to register. Even if they do not want to, I can convince them." (Shasthya Shebika*/*SS, BRAC, KII 7)*

"We have been working in EPI for 25–30 years, so people have trust in us.” (HA, Govt., KII 43).

### Provision of ‘free of cost’ materials and telemedicine services

The free-of-cost materials like masks and soaps and telemedicine services helped engage the community and increase their awareness and satisfaction. According to all the respondents, people, especially the poor, appreciated the free masks, soaps, and handwashing stations. The free materials protected the people from COVID-19 and motivated them to change their behaviors.

*“From BRAC, mask and soap were distributed to people particularly among the poorest. This is effective for motivating people to practice mask-wearing and washing hands with soap.” (SK, BRAC, KII 19)*

The rural communities also highly appreciated the telemedicine service because of the scarcity of qualified doctors. It also saved travel costs and lessened people’s fear of COVID-19.

“The best part of the project is quick communication with the MBBS doctor through telemedicine service; it is saving time and money for the poor people.” (SK, BRAC, KII 21)

*“If you want to consult a good doctor, you have to pay a 500-taka fee, as well as the travel expenses. But here people are getting treatment from a good doctor at home and getting recovered.” (SK, BRAC, KII 21)*

*“Telemedicine was a timely initiative. It was beneficial to the people, and people were less scared of COVID-19. Many misconceptions were removed from people. It will also reduce pressure from hospitals.” (Telemedicine doctor, BRAC, KII 52)*

### Confidence and motivation of the CCPC members to go beyond the assigned tasks

Several respondents identified the confidence and motivation of CCPC members as important facilitators. The CCPC members themselves also echoed it during the survey. Fifty-seven percent of the CCPC members perceived they were well-prepared to perform their assigned tasks and reported the benefits of joining the committee. Despite the understanding that their roles are voluntary, more than half (55.19%) of the CCPC members were dedicated to joining the committee to fight the COVID-19 pandemic. They wanted to contribute to the community and desired recognition. Furthermore, 93.54% of CCPC members expressed interest in continuing the tasks after the COVID-19 project ended.

### Increased demand for services from the community

Due to the people's awareness, the demand for services from health workers and community clinics also increased. More people sought vaccine registration services and were vaccinated. It also led to increased demand for case detection and telemedicine services. The implementers became accountable because people would question community health workers when they did not visit their homes. They also asked SKs for financial assistance and treatment details. Families refused SKs entrance to their homes without washing hands and wearing masks.

*“The community members ask SKs about why she did not visit home or the reason for the delay in recovering, where is the money I was supposed to get. This was a big achievement.” (Telemedicine doctor, BRAC, KII 52)*

“When I go to the house, they do not want to take the service from me without wearing a mask and washing hands.” (SK, BRAC, KII 10)

Due to the project activities, the number of patients visiting the community clinic, including pregnant mothers and children, increased and CHCPs at the CCs were also motivated to provide good services.

“The community clinic is very active. This is the best part of the project. Now I can provide more service to pregnant women and children.” (FO, BRAC, KII 23)

### Implementation barriers

#### Training need

Several key informants identified insufficient training of the CCPC and CST members as a barrier to performing their tasks in the COVID-19 project. The CCPC and CST members also opined similarly during the survey. Forty-three percent of the CCPC members and 27% of the CST members reported that although they received orientation on the COVID-19 project before implementation started, that one-day training was not enough for them. There were many gaps and disparities in the respondents' knowledge about the project activities, their roles and responsibilities, and the responsibilities of other actors in the project as well. The SKs were required to use Tab and software for entering data in their daily work and reported their lack of technological skills and knowledge. Several SKs also had trouble using devices like the pulse oximeter and infrared thermometer. The need for capacity building was especially highlighted for improving CST members’ technological skills and their knowledge about COVID-19.

*“The technological sides of the app-related things need to be simplified, and the field workers (SKs) need to be trained more on this. Then the work would be easier.” (Telemedicine doctor, BRAC, KII 54)*

### Irregular participation of the CCPC members

Several respondents reported irregular participation of the CCPC members in monthly meetings and COVID-19 project-related activities. At the community level, ensuring the attendance of all CCPC members was challenging. CCPC members were busy with their professions or other commitments. As a result, many would not attend the CCPC meetings regularly and were too busy or reluctant to give time for the project activities. Attendance was further diminished during harvest or election season.

“All the members of the village are hardworking people. So, it was difficult to organize meetings during working hours…Not everyone can be present.” (FO, BRAC, KII 32)

In a few CCs, some of the CCPC members selected were not aware that they were in the committee and hence were not active members.

### Favouritism of CCPC members in distributing masks, soaps, and handwashing stations

BRAC provided cloth masks, soaps, and handwashing stations for the disadvantaged members of the community. The CCPC members were engaged in making a list of the poor and ultra-poor for this distribution. Several CCPC members included their friends and relatives on the list, and influential members like other UP members and CHCPs claimed materials for themselves. According to respondents, several powerful community members asserted that they would not allow the distribution of materials unless given a share of the products first. As a result, some ineligible people were added to the list and received the materials.

*"The CCPC committee was told to list of 150 poor people in the area. Community members thought UP members were distributing, so they asked for more. The members themselves asked for their people." (FO, BRAC, KII 1)*

“There was a challenge to select the 30 from 200. Most of the people selected turned out to be people recommended by the Union Parishad member.” (HA, Govt., KII 43)

### Disruption in supply and shortage of the materials (masks, soaps, handwashing stations)

The distribution of materials was delayed because of the late signing of the project agreement and thus fund release which was aggravated by the nationwide lockdown. That’s why the products arrived at the sites in small batches and separately, not all components of the package at a time based on procurement delay. For example, in Narayanganj, the handwashing stations arrived, but the soaps came later. In Bogura, the masks and soaps did not arrive simultaneously. These delays created additional challenges in the distribution of the materials. Once masks were given to certain people, it was tough to gather them again at the CC to distribute the soaps.

*“We received the masks and the handwashing stations but not the soaps. We have already distributed the masks and the handwashing stations, and later we got the soap. Now, what will we do with the soap? Who will we give it to? How will we find those 100 or 200 people who got the mask?... We had to call them by phone and find them again. We informed the management, but they had no solution.” (AM, BRAC, KII 9)*

“*If materials arrived early and we had enough time to prepare between receiving and distribution, the works could be managed more efficiently. Their agitation could have been better handled or avoided.” (FO, BRAC, KII 31)*

Respondents across three study sites reported not having enough materials to distribute to the poor. Implementers mentioned that many eligible poor people had to return home empty-handed due to inadequate supply. Due to a funding shortage, there were never enough supplies for all.

"If each of the 15 CCPC members lists three poor people, that is 45 people. But I could give only 15 packages in that area. So, it was not enough.” (FO, BRAC, KII 28)

*“There were not enough materials for everybody. People complained. Say, I got 24 handwashing stations for one CC. I gave it to the poorest of the poor. But even the number of poor people was 200 out of the 6000 population. So, it was not possible to give everyone.” (AM, BRAC, KII 9)*

The shortage of materials caused dissatisfaction. The implementers faced difficulty and moral conflict when choosing between equally poor or disadvantaged people. Some dissatisfied people also threatened the service providers.

*“When people were listed, their names and other details were taken, and they automatically expected that they would receive something. So, I felt bad when we could not give them.” (FO, BRAC, KII 28)*

Another barrier was the long distance between the remote villages and the poor transportation system. Due to geographical constraints, the essential COVID-19 preventive materials for distribution took much longer than the average, especially on islands like Monpura of Bhola sub-district.

### Improper use of handwashing station and lack of follow-up

Respondents reported that many users kept the handwashing stations unopened or used them to store drinking water. There was confusion about using the handwashing station in the household. A total of 96 handwashing stations were observed, and only 55 handwashing stations were set up, while the remaining were found to be intact, unopened packages, stored away, or missing from the expected locations. Among the set-up handwashing stations, 39/55 had water, and only 25 had soap present during the visit. Some implementers expressed doubts about the proper usage of the handwashing stations because they did not have a monitoring system. There was also a risk of the handwashing station being stolen if kept outside. So, the people chose to keep it inside their homes which reduced community access to the station.

“BRAC should follow up on the usage of handwashing stations, whether they are being used, or being stored somewhere.” (HA, Govt., KII 43)

“We are giving it to people who know nothing about handwashing. It would have been better if there was a monitoring system to monitor how they are using it.” (AM, BRAC, KII 9)

### The non-compliant attitude of the community toward COVID-19 protective measures

Implementers reported difficulty ensuring compliance (especially in remote areas) regarding the three critical COVID-19 protective measures: mask-wearing, handwashing, and social distancing. Most people only wore masks when entering banks or other office buildings and were reluctant to follow the advice. Others complained about difficulty breathing while wearing masks and not having the money to buy them.

Most rural people practiced washing their hands without soap and thought only water was enough. Many people also could not afford to buy soap.

“The usual practice over here is people use soap to wash hands after eating, not before.” (HA, Govt., KII 43)

*“Community members said, “Listen. For us, the rice is finished before getting the salt (Nun aante panta furay). Where will we get soap from every day? What will we wash our hands with? What will we use?” (SS, BRAC, KII 4)*

### Challenges of ensuring home quarantine and distributing financial support for eligible COVID-19-positive cases

BRAC SKs identified suspected COVID-19 patients who needed to be in-home quarantine for 14 days. Poor or ultra-poor could receive 2000 Bangladeshi Taka to support their expenses during this period. They were also linked with telemedicine services for further follow-ups.

However, it was challenging for the implementers to ensure the home quarantine. Most people left their homes and moved almost immediately after their symptoms improved. Many went out to buy food or collect money for their families in absence of alternative options. Several families could not afford to spare a room for the quarantined suspected case as per recommendations.

*“If families had two rooms for five people, giving one room for quarantine is impossible. If they have a child, they will not stay without supporting the children. So, the social and economic environment for maintaining quarantine is not there.” (Telemedicine doctor, BRAC, KII 52)*

Several community members did receive financial support on time. There were cases when people feigned COVID-19 symptoms to receive financial support.

*"Sometimes people say they have fever even if they do not have it so they can get 2000 Bangladeshi Taka. So, the SKs call us for help. Or they talk to the doctor, and the doctor can determine if the symptoms are real." (HA, Govt., KII 43)*

Another challenge was tracking home quarantine patients for disbursement of financial support. The SK at the field regularly needed communication with the Area Manager about the number of suspected cases identified.

### Challenges regarding telemedicine and other technology issues

The study revealed a few barriers in the telemedicine service's communication, diagnosis, and follow-up processes. Firstly, some respondents reported that call waiting time was long during telemedicine service. Secondly, telemedicine doctors reported that it was often difficult to diagnose the patients over the phone as sometimes the patient showed symptoms that overlapped with other diseases. In addition, the pathological testing needed for a robust diagnosis was usually unavailable at the patients' locations. Lastly, people with no cell phones were difficult to follow up. Doctors mentioned that as some patients used their neighbour’s or relative's phones for the telemedicine service, it was challenging to further follow up.

*“Most remote people do not have mobile phones. Many people use their neighbour's or relative's phone. So, we would not find them in follow-up calls.” (Telemedicine Doctor, BRAC, KII 54)*

Most of the respondents reported having network interruptions resulting in delayed patient data transmissions and difficulties in calling the telemedicine doctors. It was also a problem during vaccine registration at the household level.

“Often due to the poor internet connection, I have to write down the person’s information in a diary and then do the registration later.” (SK, BRAC, KII 16)

The field workers’ devices (i. e. tablet computers, pulse oximeters, digital thermometers) would occasionally malfunction or give erroneous data. Because of the outage in the electricity supply, the tablets could not be charged.

### Lack of coordination among stakeholders

There were difficulties in coordination among the project stakeholders at various implementation steps. As the project activities were primarily CC-based, access to the CC was crucial for implementing the project. It required coordination between the Government and BRAC. Coordination was challenging as few government health care providers (CHCPs) considered the project activities inconvenient as their work schedule did not match that of the BRAC community health workers. According to respondents, several CHCPs felt BRAC staff monitored their work and would eventually replace them.

Due to these perceptions, many CHCPs were reluctant to collaborate. One BRAC senior manager reported that although coordination was present between the Government and BRAC at the central level, a more systematic effort for coordination was required in the field.

### The short duration of the project

Most respondents mentioned that the project duration was insufficient to train all the implementers and complete all the objectives while maintaining high standards. One SK said that the project period was almost over, but the vaccine registration volunteers had not covered all the villages in their catchment area yet. The short duration of the project was also a challenge for the awareness-raising component of the project, as people generally require more time to retain the information.

“The project duration was too short for all the activities undertaken.’” (Senior management, BRAC, KII 50)

The amount of work assigned was also significant compared to the time allotted. Respondents mentioned that it was difficult for them to plan and complete their tasks in such a short time.

*“Sometimes I was told to finish 14 meetings within four days. It was a big challenge. The CCPC committee members are busy with other work too, so I needed at least two days to tell them.” (FO, BRAC, KII 28)*

## Discussion

Our study is insightful in understanding the experience of an initiative against COVID-19 that engaged the community in delivering the program. Building the capacity of the community members is the first step of community engagement, and both CCPC and CST members demonstrated a high level of knowledge. The major success of the program is accessing underprivilege populations through community engagement and telemedicine, which otherwise might not have been possible. The trained CCPC and CST members informed, distributed COVID-19 preventive materials, and referred symptomatic patients for appropriate care.

Major enablers for the program were the engagement of diverse and influential community members, greater acceptability of the front-line health workers, distribution of free-of-cost materials, and availability of telemedicine services. However, the project struggled due to several salient barriers, including irregular participation of the CCPC members, favouritism of the CCPC members in distributing the essential COVID-19 preventive materials, disruption in the essential COVID-19 preventive materials supply, network interruptions, lack of coordination, the short project duration.

Community engagement can become imperative during the programme implementation [18] as it enables an informed implementation process incorporating the knowledge and feedback from the concerned community [19] Community engagement was also constantly emphasized in the WHO’s Strategic Preparedness and Response Plan for COVID-19 [20] and strong community participation is required to build community resilience against COVID-19 or any other pandemic emergency. It is also considered an effective mechanism for universal health coverage [21].

Among different pathways of emerging community resilience initiatives, the BRAC COVID-19 project could be categorized as a ‘Hierarchical Pathway: initiatives by external actors’ (BRAC) where the community members work as volunteers [22]. The BRAC COVID-19 project formed a bridge between the community and the providers via CCPC development, which boosted community confidence in the project’s operations.

Moreover, this initiative capitalised on the community’s established trust and good perception of BRAC and government health personnel, allowing for easier access to the population and achieving the project goals. Another enabler was having a group of confident and devoted CCPC members prepared to contribute beyond their allocated roles. Community involvement relied on a bottom-up approach in the program’s decision-making processes, such as member selection and formation of the CCPC committee and listing the poor and ultra-poor for essential COVID-19 preventive material distribution. Evidence suggests that although the participatory approach of program implementation following both the top-down and bottom-up models has its benefits and drawbacks, this partnership model of implementation [23] falls in the middle and attempts to gain from both benefits.

The provision of telemedicine services might provide long-term benefits beyond adaptive resilience by improving structures and methods of operation to meet COVID-19-related changes better and produce systems that are more fit for the new circumstances. Utilizing telemedicine services for COVID-19 could partially reduce the additional burden (due to the emergence of COVID-19) of the healthcare system to ensure the continuation of the essential healthcare services system of the country [12].

However, despite the BRAC’s programme’s importance and the presence of several facilitators, this research found various impediments to achieving community resilience. Lack of adequate training among CCPC and CST members was one. Early literature has shown that proper training may increase front-line implementers' morale, hence boosting overall implementation [24, 25]. Since CCPC members were SBCC agents in the community, they needed to be well-prepared to develop or upgrade before teaching the community. However, one of the reasons for insufficient training was the short duration of the project. Besides, it was urgent to go fast in implementation considering the quick move of the pandemic. Not all implementers knew COVID-19 symptoms, transmission methods, and prevention. Since CCPC members are more community members than formal healthcare practitioners (like HA or SK), they are less informed than CST members. This was evident in their practices related to COVID-19 prevention. Besides, most CCPC and CST members could recall two to three assigned tasks of the COVID-19 project.

Several key informants agreed, citing poor training of CCPC and CST members as a hindrance to their roles in the COVID-19 initiative. This indicates the necessity of mandatory and refresher training for the front-line implementers, ensuring better project execution. Considering the crucial role of community health workers and volunteers in promoting adaptive resilience during infectious disease outbreaks, investing in them will improve community health capacity and preparedness for future pandemic situations [26].

Due to late procurement and transport, the supply of essential COVID-19 preventive materials was disrupted throughout this project's deployment (masks, soap, handwashing stations). The reasons for disruption were delay in fund release, delay in procurement, delay in transport due to lockdown leading to a lack of supplies, and a delay in delivery in the community. While essential COVID-19 preventive materials can link the community and CCPC members for the BRAC's project, the interruption may have hampered implementations, fostering dissatisfaction and mistrust. Earlier research from other public health programs indicated that establishing the connection between the community and service providers may be a pillar to supporting a public health intervention [27].

The project’s short length hampered the implementation process significantly. Each project contains planning, procurement, recruiting, and employee training stages. Due to the project’s short duration, providing adequate training for all implementers and excellent completion of all operations was difficult. Moreover, implementers had to execute all jobs quickly, increasing their burden. Adding a year to the project’s lifespan would significantly boost its success and effect. Future studies should examine fidelity and adjustments over time to determine whether they produce resilience. Moreover, building community resilience may need a multi-sectoral approach [28].

Long call waiting time during telemedicine services was also cited by most respondents, indicating greater demand for the service than currently delivered. Also, telemedicine clinicians struggle to diagnose COVID-19 over the phone because testing facilities are required for a correct diagnosis. People without phones were difficult to contact for follow-up. The poor network also hampered implementation, especially in rural regions. Other research has revealed similar obstacles to efficient telemedicine service rollout in Bangladesh [29].

The learning from this study is to connect the activities of the various implementers by building effective horizontal communication channels between them and the community they would serve. Community engagement initiatives should draw on local infrastructure and be mindful of the heterogeneity of the community and the support they need to ensure the quality of the program. Hence, policymakers and implementers should consider rigorous training with regular refreshers, uninterrupted logistic supply, coordination among stakeholders, context, and a longer intervention period for successful community engagement to create a resilient community against COVID-19.

### Strengths and limitations

This is the first study in Bangladesh that represents the COVID-19 mitigation implementation process and associated factors. The mixed-method design allowed scope for data triangulation. There are a few limitations. A significant portion of the project period was under strict restrictions like nationwide lockdown, limiting the data collectors' movement. There is a probability of information bias as methods of collecting information were not the same for all participants (face-to-face vs. via telephone or online).

## Conclusions

Community engagement with health services through Government-NGO partnership is an essential strategy for implementing community-based projects. The study findings suggest that the community-based approach established a link between implementers and beneficiaries and facilitated engagements and activities at the grassroots level to implement the COVID-19 mitigation project. It created a window of opportunity for collaboration between community members and project implementers, laying the groundwork for a more resilient partnership. However, engagement structures should incorporate a systems perspective to facilitate the relationships, ensure the quality of the delivery program, and be mindful of the heterogeneity of different stakeholders concerning capacity building. Taking lessons from past initiatives adopted by HIV/AIDS or Ebola fighting organizations can help achieve a robust community engagement initiative/plan to stop the spread of COVID-19 [30]. Reaching out to the underprivileged and hard-to-reach populations through community engagement is also an effective mechanism to progress through universal health coverage [21].

## Supplementary Information

**Additional file 1.**

**Additional file 2.**

**Additional file 3.**

## Data Availability

The dataset generated and/or analyzed during the current study is not publicly available due to the potential risk of identification of participants, but a limited dataset is available from the corresponding author on reasonable request.

## Abbreviations

AM: Area Manager
ANC: Antenatal Care
CC: Community Clinic
CCPC: Community Corona Protection Committee
CHCP: Community Health Care Provider
CST: Community Support Team
DM: Divisional Manager
FO: Field Organizer
FWA: Family Welfare Assistant
HA: Health Assistant
HNPP: Health, Nutrition, and Population Programme
IPC: Infection Prevention and Control
IRB: Institutional Review Board
KII: Key Informant Interview
SBCC: Social and Behavioral Change Communication
SK: *Shasthya Kormi*
SS: *Shasthya Shebika*
WHO: World Health Organization
