# Supplementary material for: Implementation barriers and facilitators to a COVID-19 intervention in Bangladesh: The benefits of engaging the community for the delivery of the programme
Source: BMC Health Serv Res. 2022 Dec 28;22:1590. doi: 10.1186/s12913-022-08939-7 (PMC9795148; doi:10.1186/s12913-022-08939-7)
Supplement: Supplementary file 1 — Additional file 1. [file 12913_2022_8939_MOESM1_ESM.docx]

**Supplementary file**

*Supplementary Table 1: List of project activities to create community resilience*

| **Types of resilience capacity** | **Project activities** |
| --- | --- |
| **Adaptive resilience:** The ability of community to adjust with COVID-19 pandemic situation. | - Involving community people as implementing agent through the formation of CCPC - Capacity building (orientation/ refreshers) of the CCPC and CST members - Awareness rising at community level about preventive measures, sign symptoms, transmission through household visits and monthly meeting |
| **Absorptive resilience:** The ability to absorb and effectively cope with COVID-19; the capacity to manage and recover from adverse conditions, using available skills, assets and resources. | - Masks, soaps, and handwashing stations distribution among the poor - Ensuring home quarantine and home isolation supports for the poor (food, medicine, transportation support) - Linking to referrals (Upazila health complex and other tertiary level facilities) |
| **Anticipatory resilience:** The ability to reduce risks by means of proactive action to minimize vulnerability. | - Practice of preventive measures of COVID-19 by implementers as well as community people - Early identifying of active case of the COVID-19 |
| **Transformative resilience:** The ability of systems to transform their structures and means of operating to better address change and uncertainty due to COVID-19 and to develop (new) systems that are more suited to new conditions. | - Collaboration with local government health system - Telemedicine services |

Supplementary Table 2: Sampling strategy and sample size for the quantitative survey

| **Selection tiers** | **Sampling strategy** | **Highland** | **Plain** | **Coastal** |
| --- | --- | --- | --- | --- |
| **Districts (3)** – one from each type of geographical region | Purposive | Bogura | Narayanganj | Bhola |
| **Sub-districts (3)** – three from each district | Simple random sampling | 3 (Dupchachia, Gabtoli, Shobganj) | 3 (Araihajar, Rupganj, Sonargaon) | 3 (Monpura, Borhanuddin, Tajmuddin) |
| **Community Corona Protection Committee/CCPC (60)** | Simple random sampling | 20 | 20 | 20 |
| **Community Support Team/CST (60)** | Simple random sampling | 20 | 20 | 20 |

Supplementary Table 3: Qualitative study participants by study areas

| **Respondents (implementing agents) [N=54]** | **Bogura** | **Narayanganj** | **Bhola** |
| --- | --- | --- | --- |
| BRAC implementers at field level |  |  |  |
| Area Manager (AM) | 1 | 1 | 2 |
| Telemedicine doctor | 1 | 1 | 1 |
| Field Organizer (FO) | 4 | 4 | 3 |
| *Shasthya Kormi* (SK) | 3 | 4 | 5 |
| *Shasthya Shebika* (SS) | 3 | 3 | 5 |
| Government providers at field level |  |  |  |
| Health Assistant (HA) /Family Welfare Assistant (FWA) | 3 | 3 | 3 |
| Community Health Care Provider (CHCP) | 1 | 1 | 0 |
| BRAC Divisional Manager/DM (Central/Head Office) | 1 | 0 | 1 |
| **Total** | **17** | **17** | **20** |

Supplementary Table 4**:** Topic discussed during the training of the CCPC and CST members (only among those who received training)

| ***Indicators*** | **CCPC members** | **CST members**  **(n=68)** | |
| --- | --- | --- | --- |
| **Topics discussed in the training reported by the CCPC members (Self-reported)** | **(n=472)** | **SKs**  **n=38** | **HAs**  **n=30** |
| Signs and symptoms of COVID-19 | 354 (75.00) | 34 (89.47) | 26 (86.67) |
| COVID-19 preventive practices: Use of masks, hand washing, & social distancing | 197 (41.74) | 18 (47.37) | 12 (40.00) |
| General idea about COVID-19 and its transmission | 185 (31.19) | 27 (71.05) | 26 (86.67) |
| Ways to identify presumptive COVID-19 cases | 90 (18.33) | 8 (21.05) | 9 (30.00) |
| Available supports and services facility for COVID-19 treatment | 71 (14.46) | 6 (15.79) | 5 (16.67) |
| Testing center for COVID-19 | 67 (13.65) | 7 (18.42) | 5 (16.67) |
| Ways to arrange isolation/quarantine of positive COVID-19 cases and contacts at home | 66 (13.44) | 6 (15.79) | 2 (6.67) |
| Ways to set up cost-effective handwashing stations at HH level and in the community | 58 (11.81) | 7 (18.42) | 3 (10.00) |
| About vaccination of COVID-19 | 50 (10.59) | 6 (15.79) | 5 (16.67) |
| Making referral linkage between CST members and testing and treatment facility | 28 (5.70) | - | - |
| Creating referral link with different COVID-19 testing facility^†^ | - | 7 (18.42) | 4 (13.33) |
| Ways to measure temperature^†^ | - | 12 (31.58) | - |
| Way to measure oxygen saturation^†^ | - | 7 (18.42) | - |
| Way to categorize PII^‡^, SPSC^§^ etc. ^†^ | - | 3 (7.89) | - |

†= these activities were only applicable for CST members; ‡= Potential Infected Individuals; §= Screened Positive Suspected Case
