# Supplementary material for: Implementation barriers and facilitators to a COVID-19 intervention in Bangladesh: The benefits of engaging the community for the delivery of the programme
Source: BMC Health Serv Res. 2022 Dec 28;22:1590. doi: 10.1186/s12913-022-08939-7 (PMC9795148; doi:10.1186/s12913-022-08939-7)
Supplement: Supplementary file 2 — Additional file 2. [file 12913_2022_8939_MOESM2_ESM.docx]

**IR components: Survey tool on implementation fidelity**

Research on the “Comprehensive COVID-19 Response through Community Mobilization

and Strengthening Community Clinics”

BRAC James P Grant School of Public Health, BRAC University, Dhaka

**Respondents:** CCPC members

**Part 1: Introduction**

Greetings. I am [NAME OF INTERVIEWER], working at the BRAC James P Grant School of Public Health, BRAC University. As you know that BRAC is implementing **‘****Comprehensive COVID-19 Response through Community Mobilization and Strengthening Community Clinics’** in your areas to help improve detection and management of COVID-19 cases as-well-as reduce stigma, fear, and discrimination in the community. We are conducting a study to explore the implementation fidelity [a. adherence to protocol: content, coverage, frequency, and duration; and b. moderators: intervention complexity, facilitation strategies, quality of delivery, participant responsiveness] of this project. We would like to speak with you about your current experiences with this project implementation. Your responses and opinion will strengthen our understanding regarding this project. We hope that our study will contribute to improving/fine tuning the project implementation in the next phases.

Our conversation including the information you will share with us will be strictly confidential, and there is no risk associated to your participation in this study. Nevertheless, you are free to withdraw from this interview at any time. This interview will take around 40 minutes of your valuable time.

We may call you again in the future to see if the circumstances have changed, or to ask additional questions. Should you have any question about the study, please feel free to call us back. Please make a note of our phone number [INTERVIEWER’S CONTACT NUMBER]. If you have any further question(s) regarding the project, you can always contact us through the telephone number given below.

Do you have any questions? If not, can we start the interview? If yes, may I confirm your name and signature?

**Part 2: Identification and interview related information**

| **Sl. No.** | **Question** | **Codes/answers** | **Instruction** |
| --- | --- | --- | --- |
| surveyor | Name of the surveyor |  |  |
| date | Date of the survey |  |  |
| district | District Name | 1=Bogura  2=Narayanganj  3=Bhola |  |
| upazila | Upazilla code/Name of the Upazilla |  |  |
| union | Union code/Name of the Union |  |  |
| ccpc | Name of the CC |  |  |
| reid | Questionnaire ID: |  |  |

**Part 3: Background information of the respondent**

| **Sl. No.** | **Question** | **Codes/answers** | **Instruction** |
| --- | --- | --- | --- |
| name | Name of the respondent: |  |  |
| number | Phone number: |  |  |
| position | What is your position named in this particular project? | 1= Union Parishad member/Jomidata  2= HA  3= FWA  4= CHCP  5= Person with disability  6= SS (*Shasthya Shebikas)*  7= SK  8= headmaster/schoolteacher  9= Imam/religious leader  10= Village Doctor  11= Adolescent girl/boy  12= Muktijodhha  13= Esteemed local figure  96 = Others (specify…) |  |
| sex | Sex: | 1=Male  2=Female |  |
| age | Age: | ……………Year |  |
| educ | Educational attainment [class passed]/  What was the highest educational attainment [class passed] by the respondent? | Need to write the highest class they passed  1 = class 1,  2-9 = class 2, .........9,  10 = class 10  11 = SSC/*Dakhil*,  12 = HSC/*Alim*,  13 = diploma/vocational.  14 = BA/ BSc/BCom/*Fazil*/graduate/ BA (honours),  15 = MA/Phd/*Kamil*,  16 = *Hafezi/Qawmi/Kharizi*,  77 = N/A, because never went to school,  88 = N/A, because went to school but hasn’t passed any class;  97 = Don’t know |  |
| heard | Have you ever heard of “Comprehensive COVID-19 Response through  Community mobilization  and Strengthening Community Clinics” implemented by BRAC? | 1=Yes  0=No |  |
| rsn_join | What are the reasons that made you join the CCPC?  [Multiple answers possible] | 1= I was asked by the BRAC employees  2= I thought it would make me look like an important figure  3= I thought I can bring about positive changes in the community if I join  4= I think I have influential power in the community  5= Because I was asked politely  6= For no particular reason  7= To help fight COVID 19 in my community  96= Others (specify………………….) |  |
| continue | Do you want to continue to do the work even after the project has ended? | 1= Yes  0= No |  |
| benefit | What are the benefits for joining the CCPC?  [Multiple answers possible] | 1= People recognize me more  2= BRAC gave me additional money for doing the job  3= I get to know about the COVID-19 prevention matters way before anyone does  4= I can earn some extra cash for my labor  5= They give free food, soap and masks during the meetings  96= Others (specify………………….) |  |
| Add | Did you ever do anything to aware community people about COVID-19 in addition to what BRAC requested you to do as CCPC member? | 1= Yes  0= No |  |
| Add_yes | If yes, what are those activities? | 1= I went to households/door-to-door  2= I counselled my distant relatives by phone  96= Others (specify………..)  97 = Don’t know |  |
| Add_no | If not, why? | 1= I think my current work is enough  2= I have no extra time  3= I don’t think I have to do more tasks  96= Others (specify………..)  97 = Don’t know |  |
| commune_brac | To whom you regularly communicate regarding this BRAC COVID related project. (multiple) | 1= SK apa  2= PO/FO bhai/apa  3= Telemedicine doctor  4= Area Manager (Jannatun apa)  96= Others (specify………..)  97 = Don’t know |  |
| Rep | Do you submit any “paper” (hard copy) reports for this project? | 1=Yes  2=No |  |
| Rep2 | How many reports do you submit? | --------------------- |  |
| Rep3 | How frequently do you submit a report? | 1= monthly  2= weekly  3= bi-monthly  4= 3 monthly  5= 6 monthly  96= Others (specify………..)  97 = Don’t know |  |

**Part 4: Knowledge and practice about COVID 19**

| **Sl. No.** | **Question** | **Codes/answers** | **Instruction** |
| --- | --- | --- | --- |
| c1 | Have you ever heard of the novel coronavirus? | 1=Yes  2=No |  |
| c2 | Have you ever heard of COVID-19? | 1=Yes  2=No |  |
| C1_1 | Do you think there is a difference between coronavirus and covid-19? | 1=Yes  2=No |  |
| C1_2 | What is the difference between these two terms? | 1= Coronavirus is a virus  2= COVID-19 is the disease  96= Others (specify…….) |  |
| c3 | Tell me about the modes of the novel coronavirus transmission.  [Multiple answers possible] | 1= Droplets from coughing and sneezing  2= Touching other people who have the virus  3= Through handling contaminated money  4= Touching contaminated surfaces  5= Touching your eyes, nose or mouth with unclean hands  6= Body fluids from an infected person  7= Feces of an infected person  8= Through air  96= Others (specify………..)  97 = Don’t know |  |
| c4 | Do you think that people affected by the new coronavirus always reveal symptoms? | 1= Yes, always reveal symptoms  2= No, not always reveal symptoms  97 = Don’t know |  |
| c5 | Tell me the common symptoms of the novel coronavirus infection or COVID-19  [Multiple answers possible] | 1=Fever or chills  2=Cough  3=Fatigue  4=Muscle or body aches  5= Shortness of breath or difficulty breathing  6=Congestion or runny nose  7=Sore throat  8=New loss of taste or smell  9=Headache  96= Others (specify)  97=Don't know |  |
| c6 | How can you prevent the transmission of novel coronavirus?  [Multiple answers possible] | 1= Regularly and thoroughly clean your hands with an alcohol-based hand rub or wash them with soap and water  2= Maintain distance from other people  3= Maintain at least 1-meter (3 feet) distance from other people  4= Avoid touching eyes, nose and mouth  5= Covering mouth and nose with bent elbow or tissue while coughing or sneezing  6= Staying at home  7= Stay home and self-isolate even if you have minor symptoms such as cough, headache, mild fever, until you recover  8= Wearing face mask while going out  9= Avoid the 3Cs: spaces that are closed, crowded or involve close contact  10= Cleaning and disinfecting surfaces frequently especially those which are regularly touched, such as door handles, faucets and phone screens.  11= If you have a fever, cough and difficulty breathing, seek medical attention immediately  96= Others (specify)  97=Don't know |  |
| c7 | Describe (and demonstrate) the proper hand washing technique  [Multiple answers possible] | 1= Wet hands with water  2=Apply enough soap to cover all hand surfaces  3=Rub hands palm to palm  4=Right palm over left dorsum with interlaced fingers and vice versa  5=Palm to palm with fingers interlaced  6=Backs of fingers to opposing palms  with fingers interlocked  7=Rotational rubbing of left thumb clasped in right palm and vice versa  8=Rotational rubbing, backwards and forwards with clasped fingers of right hand in left palm and vice versa  9=Rinsing both hands with water  10=Scrub hands for at least 20 seconds  96=Others (specify)  97=Don’t know |  |
| c8 | In what cases, do you clean your hands using soap and water to prevent novel coronavirus transmission?  [Multiple answers possible] | 1=After coming from outside home  2=Before touching eyes, nose, and mouth  3=Before prepare food  4=Before eating  5=After touching a surface or object that may be frequently touched by other people, such as door handles, tables, remote controller, button/switch, money, etc.  6=After blowing nose, coughing, or sneezing into hands  7=Before and after caring for someone who is sick  8=After using the toilet  9=After touching an animal, animal feed, or animal waste  10=After touching garbage  11=Do not use soap at all  96=Others (Specify)  97=Don’t know |  |
| c9 | What are the criteria for proper mask-wearing?  [Multiple answers possible] | 1=Verify which side is the top – this is usually where the metal strip is  2=Identify the inside of the mask, which is usually the white side  3=Place the mask on your face covering your nose, mouth and chin, making sure that there are no gaps between your face and the mask  4=Pinch the metal strip so it moulds to the shape of your nose  5=Avoid touching the mask before cleaning hands  6=Not sharing your mask with others  7=Cleaning hands before putting mask on, as well as before and after taking it off  96=Other (Specify)  97=Don't know |  |
| c10 | How often do you wear a face mask while going out of your house? | 1=I always wear a face mask  2=I sometimes wear a face mask  3=I never wear a face mask |  |
| C10_1 | How often do your family members wear a face mask while going out of your house? | 1=they always wear a face mask  2=they sometimes wear a face mask  3=they never wear a face mask  96=Other (Specify)  97=Don't know |  |
| c11 | What do you understand by physical distancing?  [If mentions distance, ask to define it] | 1=Maintaining a distance of at least 3 feet or 1-meter from each other  96=Other (Specify)  97=Don't know |  |
| c12_c13 | In the last 2 weeks, how many times did you do these…? (See below) |  |  |
| c12 | a) Join social gatherings (e.g. market, religious gathering, wedding, etc.) with people other than your household members | Number of times in the past 2 weeks |  |
| c13 | b) Use public transport (e.g. bus, tempo, shared auto-rickshaw, train, etc.) | Number of times in the past 2 weeks |  |
| c14 | In the last 2 weeks, how often do you think you stayed at least 3 feet or 1-metre (1.3 arms-length) away from other people? | 1=Never  2=Seldom  3=Some of the time  4=Most of the time  5= Always |  |
| c15 | Do you know about the hotline or emergency number for help related to the new coronavirus infection or COVID-19?  [If ‘0’, skip to the next question] | 1= Yes  0=No | If response is ‘yes’ go to c16 & c17 |
| c16 | If yes, what is the number [let the respondent to tell the number from memory or may be from saved number in mobile] | ----------------------------- |  |
| c17 | Did you ever call any of these numbers? | 1= Yes  0= No | If response is ‘yes’ go to c18 |
| c18 | If ‘yes’, were you able to reach anyone via that number? | 1= Yes  0= No |  |
| C19 | What do you mean by “Home isolation”/quarantine?  (multiple response) | 1= Stay at home only  2= Stay at home, in a separate room from family members  3= Maintain 3 feet distance with others at home  4= Use separate clothes, bed, utensils, etc  5= Don’t allow any visitors at home  6= Wear mask at all times  7= Have other people bring food/medicines from outside  8= Cough/sneeze into tissue/cloth/elbow  9= Stay clean and wash hands with soap frequently  10= Keep commonly touched surfaces clean  97= Don’t know  96= others |  |
| C20 | Who are the people who are supposed to do home isolation? (multiple response) | 1= people with fever and cough/sore throat/lack of smell/difficulty breathing  2= people with COVID symptoms  3= People who tested positive for COVID  4= People identified by CST members (SK/HA)  97= Don’t know  96= others |  |
|  | How many days do you need to do home quarantine? | 1= 14 days  2= until there are no symptoms  3= until the doctor says so  96= Others  97= Don’t know |  |

**Part 5: About the COVID 19 project including related training and capacity building**

| **Sl. No.** | **Question** | **Codes/answers** | **Instruction** |
| --- | --- | --- | --- |
| covid_date | When were you first informed that BRAC is going to implement this project? | Month / Year |  |
| notif_medium | How were you notified about this project?  [Multiple answers possible] | 1= Through official circular (paper based)  2= Through online orientation/meeting  3= Through face-to-face orientation/meeting  96=Others (specify……) |  |
| medium | From whom?  [Multiple answers possible] | **Implementers**  1= SK  2= HA  3= PO/FO  4= Area manager (AM)  **CCPC members**  5= Union Parishad member/Jomidata  6= HA  7= FWA  8= CHCP  9= Person with disability  10= SS (*Shasthya Shebikas)*  11= headmaster/schoolteacher  12= Imam/religious leader  13= Village Doctor  14= Adolescent girl/boy  15= Muktijodhha  16= Esteemed local figure  96 = Others (specify…) |  |
| goals | What is (are) the purpose(s) of this project?  [Read out loud]  [Multiple answers possible] | 1= Reduce incidence and mortality from COVID-19 cases  2= Improve quality of life of COVID-19 cases  3= Improve access of non-COVID patients to the health services  4= Reduce stigma, fear and discrimination in community  5= Increase case detection and improve treatment outcome (cure rate).  6= Restore health services at community and upazila levels both in public and private sectors  7= Increase awareness of eligible members and their communities about vaccine, eligibility criteria  8= Increase awareness of eligible members and their communities about registration process and location of vaccination center  96= Others (specify……)  97 = Don’t know |  |
|  | **Training/Orientation of the CCPC members** | | |
| training_1 | Did you receive any training or orientation on COVID-19/ Coronavirus Prevention from BRAC HNPP program? | 1=Yes  0=No | If response is ‘yes’ go to timing_train, train_dura, times_train & topic_train |
| timing_train | When did you receive the first training or orientation? | 1=November 2020  2=December 2020  3=January 2021  4=February 2021  5=March 2021 |  |
| train_dura | Duration of the first training or orientation? | 1= 1 hour  2= 2 hours  3= Half day  4= 1 day  5= 2 days |  |
| times_train | Till date, how many training or orientation did you receive? | --------------- times |  |
| Ref_tr | Did you get any refresher trainings? | 1=Yes  0=No |  |
| Ref_trnm | If yes, How many? | ------------ times |  |
| topic_train | What are the topics covered during the training or orientation?  [multiple responses] | 1= Idea about COVID-19  2= How COVID-19 transmit/spread  3= Sign and symptoms of COVID-19  4=Hand washing and other hygiene practices  5= Use of masks  6= Social and safe physical distancing  7= Where to get COVID-19 test services  8= Other supports and services facilities  9= Identifying presumptive COVID-19 cases  10= Making referral linkage with CST for test and other services  11= Arrange isolation/quarantine of COVID-19 positive cases or their close contacts at their home  12= Arranging cost-effective hand washing stations at household and community  13= COVID 19 vaccination  96= Others (specify………………..) |  |
| Top_trbef | Did you know about any of these topics before getting the training? | 1= Yes  0= No |  |
| Top_trbef1 | If yes, which topics did you know about from before? (multiple response) | 1= Idea about COVID-19  2= How COVID-19 transmit/spread  3= Sign and symptoms of COVID-19  4=Hand washing and other hygiene practices  5= Use of masks  6= Social and safe physical distancing  7= Where to get COVID-19 test services  8= Other supports and services facilities  9= Identifying presumptive COVID-19 cases  10= Making referral linkage with CST for test and other services  11= Arrange isolation/quarantine of COVID-19 positive cases or their close contacts at their home  12= Arranging cost-effective hand washing stations at household and community  13= COVID 19 vaccination  96= Others (specify………………..) |  |
| bcc_mat1 | Have you received any BCC material from the project? [Please explain what does it mean by BCC material] | 1= Yes  0= No |  |
| bcc_mat2 | If yes, So far, what are the BCC material you received from this project?  [Please explain what does it mean by BCC material]  [Multiple response possible] | 1= Leaflets  2= Stickers  3= Module/guideline on the project  4= Poster  5= Booklet  96= Others (specify……………….) |  |
| bcc_mat3 | What Have you done with the bcc materials that you received? [Multiple response possible] | 1= Distribute in the neighbouring household  2= Distribute in the mosque  3= Distribute in the Bazar  4= Kept at my home  5= kept for my personal use  6= Hang up in a public place  96= Others |  |
| comm_mat | What is the source of information that you have on COVID 19? | 1= Mobile phone (phone-call or message)  2= Newspaper/ Poster/ Banner/ Billboard  3= TV/ Radio  4= Facebook/ YouTube/ Instagram  5= Miking  6= Family/ Relatives  7= Friends  8=Neighbours  9= Colleagues  10= Govt. CHW from CC (CHCP/ HA/ FWA)  11= Govt. CHW from FWC (i.e., MO/ SACMO/ FWV/ Pharmacist)  12=Govt. CHW from other facilities  13= BRAC CHW (i.e., SS/ SK/ PK)  14=Other NGO CHWs  15=Village-doctor/ Drug seller/ Drug store owner  16=School teacher  17= Religious leader  18=Member/ Chairman of local union parishad  96=Others (specify)  97=Don’t know |  |
| List_fam/fr/nei/col1 | (If 6, 7, 8, or 9; for each of these four options ask separately for that person being referenced), ask whether that person belongs to this list | 1= Govt. CHW from CC (CHCP/ HA/ FWA)  2= Govt. CHW from FWC (i.e., MO/ 3=SACMO/ FWV/ Pharmacist)  4=Govt. CHW from other facilities  5=BRAC CHW (i.e., SS/ SK/ PK)  96=Other NGO CHWs  6=None of them |  |
| comm_mat | As per your experiences, which one is the most useful/influencing or effective? | 1= Mobile phone (phone-call or message)  2= Newspaper/ Poster/ Banner/ Billboard  3= TV/ Radio  4= Facebook/ YouTube/ Instagram  5= Miking  6= Family/ Relatives  7= Friends  8=Neighbours  9= Colleagues  10= Govt. CHW from CC (CHCP/ HA/ FWA)  11= Govt. CHW from FWC (i.e., MO/ SACMO/ FWV/ Pharmacist)  12=Govt. CHW from other facilities  13= BRAC CHW (i.e., SS/ SK/ PK)  14=Other NGO CHWs  15=Village-doctor/ Drug seller/ Drug store owner  16=School teacher  17= Religious leader  18=Member/ Chairman of local union parishad  96=Others (specify)  97=Don’t know |  |
| List_fam/fr/nei/col2 | (If 6, 7, 8, or 9; for each of these four options ask separately for that person being referenced), ask whether that person belongs to this list | 1= Govt. CHW from CC (CHCP/ HA/ FWA)  2= Govt. CHW from FWC (i.e., MO/ 3=SACMO/ FWV/ Pharmacist)  4=Govt. CHW from other facilities  5=BRAC CHW (i.e., SS/ SK/ PK)  96=Other NGO CHWs  6=None of them |  |

**Part 6: Roles and responsibilities as a CCPC member: Community awareness component**

| **Sl. No.** | **Question** | **Codes/answers** | **Instruction** |
| --- | --- | --- | --- |
|  |  |  |  |
| ccpc_mem | Who are the members of CCPC?  [multiple responses] | 1= Union Parishad member/Jomidata  2= HA  3= FWA  4= CHCP  5= Person with disability  6= SS (*Shasthya Shebikas)*  7= SK  8= headmaster/schoolteacher  9= Imam/religious leader  10= Village Doctor  11= Adolescent girl/boy  12= Muktijodhha  13= Esteemed local figure  96 = Others (specify…)  97 = Don’t know |  |
| ccpc_task | What are your tasks as a member of CCPC?  [multiple responses] | 1= Raising awareness on protective measure, prevention, sign and symptoms of COVID-19  3= Arranging cost-effective hand washing stations, at poor/ultra-poor household and community clinics with adequate water supply and soap/detergents.  4= Distributing mask and soap to poor and ultra-poor  5= Help identify presumptive COVID-19 cases through Community Support Team (CST).  6= Making referral linkage to doctor/health facility  7= Extend food and other support to people who are undergoing home isolation/quarantine.  8= Encourage getting tested for people with COVID symptoms  9= Monitor Community Clinic/ Complete Social Audit Tool checklist  11= Encourage people to get vaccinated  12= Attend/ arrange monthly meetings  13= Make a list of the poor and ultra-poor in the community  14= accompany registered individuals for printing vaccination card  15= accompany registered individuals for vaccination  16= ensure maintenance of handwashing stations in the community clinic  96= Others (specify………….) |  |
| perform_process | What is the process you follow to perform that? (modality of service delivery)  [Multiple responses possible] | 1= community meetings  2= person-to-person/individual interactions  3= communicating with SS/SK/HA  96=Others (specify………) |  |
| Percep | How prepared do you feel to carry out the abovementioned tasks? | 1= Well prepared/ well trained  2= Averagely prepared  3= Not well prepared/ inadequately trained  96= others  97= don’t know |  |
| soap_dist | Over last 2 weeks, approximately how many soap you distributed? | ………………………………. Soap |  |
| mask_dist | Over last 2 weeks, approximately how many mask you distributed? | ………………………………. Mask |  |
| diss_inf | To whom do you disseminate the COVID 19 related protective messages?  [Multiple responses possible] | 1= Adult male  2= Adult female  3=Young male  4= Young female  5= Elderly male  6=Elderly female  96= Others (specify…………..)  97 = Don’t know |  |
| meet_attnd | Till today, did you attend any monthly meeting to discuss/share your experience, challenges, and way forward regarding this project? [monthly coordination meeting] | 1= Yes  0= No |  |
| meet_attnd2 | If yes, then how many? | _____________________ |  |
| sup | Have you ever supported anyone to do home isolation/quarantine? | 1= Yes  0= No |  |
| Supnm | If yes, How many people? | ____________________ |  |
| Howsup | If yes, how did you support them? (multiple response) | 1= got them medicine  2= got them food  3= referred them to doctor/hospital  4= Tele-counselling  96= others |  |
| Vacacc | Have you ever accompanied anyone to the BRAC vaccination assistance booth for printing vaccination card? | 1= Yes  0= No |  |
| Vacaccnm | If yes, How many people did you accompany till date? | _______________ |  |
| Travel | If yes, Did you ever get any travel/food allowance for it? | 1= Yes  0= No |  |
| Travelnm | If yes, How many times? | _______________ |  |
| Travelnmy | If yes, How much amount per visit? | ____________ |  |
| vaccin | Have you ever accompanied anyone to get vaccinated? | 1= Yes  0= No |  |
| Vaccinnm | If yes, How many people did you accompany till date? | _______________ |  |
| Vaccine_reg | How many people do you accompany for vaccination registration per day? | _______________ |  |
| Vaccinnmd | How many people do you accompany for vaccination per day? | _______________ |  |

**Part 7: Knowledge, attitude and practice on Vaccination**

| **Sl. No.** | **Question** | **Code** | **Options** |
| --- | --- | --- | --- |
| **Knowledge** | | | |
| qg_3 | Have you heard anything about a vaccine against the novel coronavirus? | 0 | No |
|  |  | 1 | Yes |
| qg_4 | (If Yes) What is the source of information that you have on vaccine? |  | 1= Mobile phone (phone-call or message)  2= Newspaper/ Poster/ Banner/ Billboard  3= TV/ Radio  4= Facebook/ YouTube/ Instagram  5= Miking  6= Family/ Relatives  7= Friends  8=Neighbours  9= Colleagues  10= Govt. CHW from CC (CHCP/ HA/ FWA)  11= Govt. CHW from FWC (i.e., MO/ SACMO/ FWV/ Pharmacist)  12=Govt. CHW from other facilities  13= BRAC CHW (i.e., SS/ SK/ PK)  14=Other NGO CHWs  15=Village-doctor/ Drug seller/ Drug store owner  16=School teacher  17= Religious leader  18=Member/ Chairman of local union parishad  96=Others (specify)  97=Don’t know |
| qg_5 | (If 6, 7, 8, or 9; for each of these four options ask separately for that person being referenced), ask whether that person belongs to this list |  | 1= Govt. CHW from CC (CHCP/ HA/ FWA)  2= Govt. CHW from FWC (i.e., MO/ 3=SACMO/ FWV/ Pharmacist)  4=Govt. CHW from other facilities  5=BRAC CHW (i.e., SS/ SK/ PK)  6=Other NGO CHWs  7=None of them |
| Qna_1 | If yes, who should get the vaccine? |  | 1= Everyone should get vaccinated  2=People over the age of 40 should get vaccinated  3=Nobody should get vaccinated  96=Others  97= Don’t know |
| qN_1 | How many BRAC COVID Vaccine assistance booths are there in this union? |  | ………… |
| qN_2 | Where can people get the vaccine from? |  | 1=CC  2=FWC  3=District hospital  4=Medical college hospital  5=Specialized hospital  6=Other public hospital/ clinic  7=Private hospital/ clinic  96=Others (specify)  97=Don’t know |
| Qna_2 | Is there any fee for getting vaccinated? (only for taking vaccine) |  | 0=No  1=Yes |
| Qna_3 | If yes, how much? |  | ---------------- |
| qN_6 | Do you know how to register for the vaccine? |  | 0=No  1=Yes |
| Qna_4 | What are the ways can you register for getting the vaccine? |  | 1=Online registration through govt. website/ app  2=In-person visit to a registration booth  3=Volunteers visit the households to register  96=Others (specify)  97=Don’t know |
| Qna_5 | Does the vaccine have any side effect? |  | 0=No  1=Yes  2=Not sure |
| qN_5 | (If yes) What are the possible side effects of the vaccine? |  | 1=Muscle pain/ body ache  2=Pain at the injection site  3=Swelling at the injection site  4=Redness at the injection site  5=Weakness/ fatigue  6=Nausea  7=Fever  8=Chills  9=Headache  96=Others (specify)  97=Don’t know |
|  | **Attitude** |  |  |
| Qna_6 | Are you willing to get vaccinated? |  | 0=No  1=Yes  3=Refuse to respond |
| Qna_7 | (If yes) Why are you willing to take it? |  | 1=To prevent COVID-19 or to prevent the novel coronavirus transmission  2=Because everyone else is having it  3=Because I have been asked to do so  4=Because It is being administered free-of-cost  96=Others (specify)  99= Refuse to respond |
| Qna_8 | (If no) Why are you not willing to take it? |  | 1=I do not think the vaccine is necessary  2=I do not know what they are giving me  3=Afraid about its side effects  4=Afraid of injections  5=Not permitted in my religion  6=The entire vaccine thing is a farce  7=There is no such thing as COVID-19 or novel coronavirus here  96=Others (specify)  99=Refuse to respond |
| Qna_9 | Are your family members willing to get vaccinated? |  | 0=No  1=Yes |
| qg_7 | If Yes: Who in your family would you prefer taking it first? |  | 1=Entire family  2=Children only  3=Pregnant  4=Elderly  5=Males of the household  6=Females of the household  7=Respondent themselves  96=Others (Specify) |
| qN_4 | Do you think that the vaccine is safe? |  | 0=No  1=Yes  3=Not sure |
|  | **Practice** |  |  |
| Qna_10 | Did you register for vaccine? |  | 0=No  1=Yes |
| Qna_11 | (If no) Why did you not register? |  | 1=I don’t want to take the vaccine  2=I don’t know how or where to register  3=I do not have internet access.  4=I don’t know the web link  5=Could not open the web link  6=Find it difficult to fill the form  7=I do not find anyone to fill out the form for me  8=I was busy  9=I do not match any of the eligibility criteria  96=Others (specify) |
| Qna_12 | If yes, how did you do it? |  | 1=Online  2=Went to a BRAC COVID Vaccination Assistance booth  3=A volunteer visited my house and did it for me  Others (specify) |
| Qna_13 | If yes, did you download a vaccine card? |  | 0=No  1=Yes |
| Qna_14 | If yes, did you print it out? |  | 0=No  1=Yes |
| Qna_15 | If yes, do you have the vaccination card? (Check to see it) |  | 0=No  1=Yes, observed  2=Yes, not observed |
| qN_10 | If yes, what are the difficulties you faced to register? |  | 1=The user interface was difficult to understand  2=Could not submit the form at first try  3=I had to manage computer/ internet access  4=I had to find someone who can fill out the form for me  5=I didn’t face any difficulties  96=Others (specify) |
| Qna_16 | Have you got the first dose of vaccine? |  | 0=No  1=Yes |
| qN_7 | Has any other member of your household register for the vaccine? |  | 0=No  1=Yes |
| qN_13 | Has any other member of your household received the first dose of vaccine? |  | 0=No  1=Yes |
| qN_14 | Did you/ any of your household members face any difficulty to get the vaccine? |  | 0=No  1=Yes |
| qN_15 | (If yes) What are the difficulties did you/ your household members face while receiving it? |  | 1=Had to travel a long distance  2=Transport cost was too high  3=Had to wait in a queue for long  4=Got some of the side effects  5=Did not take the required documents (NID, Vaccination Card)  6=Could not show the SMS sent by govt.  96=Others (specify) |
| Qna_17 | How long did it take for you to reach the centre? |  | ------------minutes |
| Qna_18 | How long did you have to wait in the queue to get the shot? |  | ______minutes |
| Qna_19 | What would be your recommendations to improve the system further? (multiple) |  | 1=Assistance in registration  2=Assistance in travelling to the centre  3=The vaccine should be made available for everyone irrespective of the eligibility  4=More vaccines should be procured  5=Should be administered to all free-of-cost  6=No recommendation  96=Others (specify) |
| qN_16 | (For those who got the first dose) Would you be willing to get the second dose? |  | 0=No  1=Yes  3=Not sure |
| Qna_20 | (If no) Why not? |  | 1=Had a bad experience while receiving the first dose  2=Got some of the side effects  3=COVID-19 or the novel coronavirus transmission is under control now  4=Do not need another shot  96=Others (specify) |

**-------------------Thank you very much for your valuable time ☺--------------------**

**IR components: Survey tool on implementation fidelity**

Research on the “Comprehensive COVID-19 Response through Community Mobilization

and Strengthening Community Clinics”

BRAC James P Grant School of Public Health, BRAC University, Dhaka

**Respondents:** CST members

**Part 1: Introduction**

Greetings. I am [NAME OF INTERVIEWER], working at the BRAC James P Grant School of Public Health, BRAC University. As you know that BRAC is implementing **‘Comprehensive COVID-19 Response through Community Mobilization and Strengthening Community Clinics’** in your areas to help improve detection and management of COVID-19 cases as-well-as reduce stigma, fear, and discrimination in the community. We are conducting a study to explore the implementation fidelity [a. adherence to protocol: content, coverage, frequency, and duration; and b. moderators: intervention complexity, facilitation strategies, quality of delivery, participant responsiveness] of this project. We would like to speak with you about your current experiences with this project implementation. Your responses and opinion will strengthen our understanding regarding this project. We hope that our study will contribute to improving/fine tuning the project implementation in the next phases.

Our conversation including the information you will share with us will be strictly confidential, and there is no risk associated to your participation in this study. Nevertheless, you are free to withdraw from this interview at any time. This interview will take around 40 minutes of your valuable time.

We may call you again in the future to see if the circumstances have changed, or to ask additional questions. Should you have any question about the study, please feel free to call us back. Please make a note of our phone number [INTERVIEWER’S CONTACT NUMBER]. If you have any further question(s) regarding the project, you can always contact us through the telephone number given below.

Do you have any questions? If not, can we start the interview? If yes, may I confirm your name and signature?

**Part 2: Identification and interview related information**

| **Sl. No.** | **Question** | **Codes/answers** | **Instruction** |
| --- | --- | --- | --- |
| surveyor | Name of the Surveyor |  |  |
| date | Date of the survey |  |  |
| district | District Name | 1=Bogura  2=Narayanganj  3=Bhola |  |
| upazila | Upazilla code/Name of the Upazilla |  |  |
| union | Union code/Name of the Union |  |  |
| ccpc | Name of the CC |  |  |
| reid | Questionnaire ID: |  |  |

**Part 3: Background information of the respondent**

| **Sl. No.** | **Question** | **Codes/answers** | **Instruction** |
| --- | --- | --- | --- |
| name | Name of the respondent: |  |  |
| number | Phone number: |  |  |
| position | What is your position named in this particular project? | 1= SK  2= HA  96=Other _____ |  |
| sex | Sex: | 1=Male  2=Female |  |
| age | Age: | ……………Year |  |
| educ | Educational attainment [class passed]/  What was the highest educational attainment [class passed] by the respondent? | Need to write the highest class they passed  1 = class 1,  2-9 = class 2, .........9,  10 = class 10  11 = SSC/*Dakhil*,  12 = HSC/*Alim*,  13 = diploma/vocational;  14 = BA/ BSc/BCom/*Fazil*/graduate/ BA (honours),  15 = MA/Phd/*Kamil*,  16 = *Hafezi/Qawmi/Kharizi*,  77 = N/A, because never went to school,  88 = N/A, because went to school but hasn’t passed any class;  97 = Don’t know |  |
| heard | Have you ever heard of “Comprehensive COVID-19 Response through Community Mobilization and Strengthening Community Clinics” implemented by BRAC? | 1=Yes  0=No |  |
| rsn_join | What are the reasons that made you join the CST?  [Multiple answers possible] | 1= I was asked by the BRAC employees  2= I thought it would make me look like an important figure  3= I thought I can bring about positive changes in the community if I join  4= I think I have influential power in the community  5= Because I was asked politely  6= For no particular reason  96= Others (specify………………….) |  |
| continue | Do you want to continue to do the work even after the project has ended? | 1= Yes  0= No |  |
| benefit | What are the benefits for joining the CST?  [Multiple answers possible] | 1= People recognize me more  2= BRAC gave me additional money for doing the job  3= I get to know about the COVID-19 prevention matters way before anyone does  4= I can earn some extra cash for my labor  5= They give free food, soap and masks during the meetings  96= Others (specify………………….) |  |
|  | Did you ever do anything to aware community people about COVID-19 in addition to what BRAC requested you to do as CST member? | 1= Yes  0= No |  |
|  | If yes, what are those activities? | 1= I went to additional households  2= I counselled my distant relatives by phone  96= Others (specify………..)  97 = Don’t know |  |
|  | If not, why? | 1= I think this is enough  2= I have no extra time  3= I don’t think I have to do more  96= Others (specify………..)  97 = Don’t know |  |
| commune_brac | To whom you regularly communicate regarding this BRAC COVID related project. (multiple) | 1= SK apa  2= PO/FO bhai/apa  3= Telemedicine doctor  4= Area Manager  5= SS  6= HA  96= Others (specify………..)  97 = Don’t know |  |
|  | Do you submit any “paper” (hard copy) reports for this project? | 1=Yes  2=No |  |
|  | How many reports do you submit? | --------------------- |  |
|  | How frequently do you submit a report? (multiple) | 1= monthly  2= weekly  3= bi-monthly  4= 3 monthly  5= 6 monthly  96= Others (specify………..)  97 = Don’t know |  |

**Part 4: Knowledge and practice about COVID 19**

| **Sl. No.** | **Question** | **Codes/answers** | **Instruction** |
| --- | --- | --- | --- |
| c1 | Have you ever heard of the novel coronavirus? | 1=Yes  2=No |  |
| c2 | Have you ever heard of COVID-19? | 1=Yes  2=No |  |
|  | Do you think there is a difference between coronavirus and covid-19? | 1=Yes  2=No |  |
|  | What is the difference between these two terms? | 1= Coronavirus is a virus  2= COVID-19 is the disease  96= Others (specify…….) |  |
| c3 | Tell me about the modes of the novel coronavirus transmission.  [Multiple answers possible] | 1= Droplets from coughing and sneezing  2= Touching other people who have the virus  3= Through handling contaminated money  4= Touching contaminated surfaces  5= Touching your eyes, nose or mouth with unclean hands  6= Body fluids from an infected person  7= Feces of an infected person  8= Through air  96= Others (specify………..)  97 = Don’t know |  |
| c4 | Do you think that people affected by the new coronavirus always reveal symptoms? | 1= Yes, always reveal symptoms  2= No, not always reveal symptoms  97 = Don’t know |  |
| c5 | Tell me the common symptoms of the novel coronavirus infection or COVID-19  [Multiple answers possible] | 1=Fever or chills  2=Cough  3=Fatigue  4=Muscle or body aches  5= Shortness of breath or difficulty breathing  6=Congestion or runny nose  7=Sore throat  8=New loss of taste or smell  9=Headache  96= Others (specify)  97=Don't know |  |
| c6 | How can you prevent the transmission of novel coronavirus?  [Multiple answers possible] | 1= Regularly and thoroughly clean your hands with an alcohol-based hand rub or wash them with soap and water  2= Maintain distance from other people  3= Maintain at least 1-meter (3 feet) distance from other people  4= Avoid touching eyes, nose and mouth  5= Covering mouth and nose with bent elbow or tissue while coughing or sneezing  6= Staying at home  7= Stay home and self-isolate even if you have minor symptoms such as cough, headache, mild fever, until you recover  8= Wearing face mask while going out  9= Avoid the 3Cs: spaces that are closed, crowded or involve close contact  10= Cleaning and disinfecting surfaces frequently especially those which are regularly touched, such as door handles, faucets and phone screens.  11= If you have a fever, cough and difficulty breathing, seek medical attention immediately  96= Others (specify)  97=Don't know |  |
| c7 | Describe the proper hand washing technique  [Multiple answers possible] | 1= Wet hands with water  2=Apply enough soap to cover all hand surfaces  3=Rub hands palm to palm  4=Right palm over left dorsum with interlaced fingers and vice versa  5=Palm to palm with fingers interlaced  6=Backs of fingers to opposing palms  with fingers interlocked  7=Rotational rubbing of left thumb clasped in right palm and vice versa  8=Rotational rubbing, backwards and forwards with clasped fingers of right hand in left palm and vice versa  9=Rinsing both hands with water  10=Scrub hands for at least 20 seconds  96=Others (specify)  97=Don’t know |  |
| c8 | In what cases, do you clean your hands using soap and water to prevent novel coronavirus transmission?  [Multiple answers possible] | 1=After coming from outside home  2=Before touching eyes, nose, and mouth  3=Before prepare food  4=Before eating  5=After touching a surface or object that may be frequently touched by other people, such as door handles, tables, remote controller, button/switch, money, etc.  6=After blowing nose, coughing, or sneezing into hands  7=Before and after caring for someone who is sick  8=After using the toilet  9=After touching an animal, animal feed, or animal waste  10=After touching garbage  11=Do not use soap at all  96=Others (Specify)  97=Don’t know |  |
| c9 | What are the criteria for proper mask-wearing?  [Multiple answers possible] | 1=Verify which side is the top – this is usually where the metal strip is  2=Identify the inside of the mask, which is usually the white side  3=Place the mask on your face covering your nose, mouth and chin, making sure that there are no gaps between your face and the mask  4=Pinch the metal strip so it moulds to the shape of your nose  5=Avoid touching the mask before cleaning hands  6=Not sharing your mask with others  7=Cleaning hands before putting mask on, as well as before and after taking it off  96=Other (Specify)  97=Don't know |  |
| c10 | How often do you wear a face mask while going out of your house? | 1=I always wear a face mask  2=I sometimes wear a face mask  3=I never wear a face mask |  |
|  | How often do your family members wear a face mask while going out of your house? | 1=they always wear a face mask  2=they sometimes wear a face mask  3=they never wear a face mask  96=Other (Specify)  97=Don't know |  |
| c11 | What do you understand by physical distancing?  [If mentions distance, ask to define it] | 1=Maintaining a distance of at least 3 feet or 1-meter from each other  96=Other (Specify)  97=Don't know |  |
| c12_c13 | In the last 2 weeks, how many times did you do these…? (See below) |  |  |
| c12 | a) Join social gatherings (e.g. market, religious gathering, wedding, etc.) with people other than your household members | Number of times in the past 2 weeks |  |
| c13 | b) Use public transport (e.g. bus, tempo, shared auto-rickshaw, train, etc.) | Number of times in the past 2 weeks |  |
| c14 | In the last 2 weeks, how often do you think you stayed at least 3 feet or 1-metre (1.3 arms-length) away from other people? | 1=Never  2=Seldom  3=Some of the time  4=Most of the time |  |
| c15 | Do you know about the hotline or emergency number for help related to the new coronavirus infection or COVID-19?  [If ‘0’, skip to the next question] | 1= Yes  0=No | If response is ‘yes’ go to c16 & c17 |
| c16 | If yes, what is the number [let the respondent to tell the number from memory or may be from saved number in mobile] | ----------------------------- |  |
| c17 | Did you ever call any of these numbers? | 1= Yes  0= No | If response is ‘yes’ go to c18 |
| c18 | If ‘yes’, were you able to reach anyone via that number? | 1= Yes  0= No |  |
|  | How should you do “Home isolation”/quarantine?  (multiple response) | 1= Stay at home only  2= Stay at home, in a separate room from family members  3= Maintain 3 feet distance with others at home  4= Use separate clothes, bed, utensils, etc  5= Don’t allow any visitors at home  6= Wear mask at all times  7= Have other people bring food/medicines from outside  8= Cough/sneeze into tissue/cloth/elbow  9= Stay clean and wash hands with soap frequently  10= Keep commonly touched surfaces clean  97= Don’t know  96= others |  |
|  | Who are the people who are supposed to do home isolation? (multiple response) | 1= people with fever and cough/sore throat/lack of smell/difficulty breathing  2= people with COVID symptoms  3= People who tested positive for COVID  4= People identified by CST members (SK/HA)  97= Don’t know  96= others |  |
|  | How many days do you need to do home quarantine? | 1= 14 days  2= until there are no symptoms  3= until the doctor says so  96= Others  97= Don’t know |  |

**Part 5: About the COVID 19 project including related training and capacity building**

| **Sl. No.** | **Question** | **Codes/answers** | **Instruction** |
| --- | --- | --- | --- |
| covid_date | When were you first informed that BRAC is going to implement this project? | Month / Year |  |
| notif_medium | How were you notified about this project?  [Multiple answers possible] | 1= Through official circular (paper based)  2= Through online orientation/meeting  3= Through face-to-face orientation/meeting  96=Others (specify……) |  |
| medium | From whom?  [Multiple answers possible] | **Implementers**  1= SK  2= HA  3= PO/FO  4= Area manager (AM)  **CCPC members**  5= Union Parishad member/Jomidata  6= HA  7= FWA  8= CHCP  9= Person with disability  10= SS (*Shasthya Shebikas)*  11= headmaster/school teacher  12= Imam/religious leader  13= Village Doctor  14= Adolescent girl/boy  15= Muktijodhha  16= Esteemed local figure  96 = Others (specify…) |  |
| goals | What is (are) the purpose(s) of this project?  [Read out loud]  [Multiple answers possible] | 1= Reduce incidence and mortality from COVID-19 cases  2= Improve quality of life of COVID-19 cases  3= Improve access of non-COVID patients to the health services  4= Reduce stigma, fear and discrimination in community  5= Increase case detection and improve treatment outcome (cure rate).  6= Restore health services at community and upazila levels both in public and private sectors  7= Increase awareness of eligible members and their communities about vaccine, eligibility criteria  8= Increase awareness of eligible members and their communities about registration process and location of vaccination center  96= Others (specify……)  97 = Don’t know |  |
|  | **Training/Orientation of the CST members** | | |
| training_1 | Did you receive any training or orientation on COVID-19/ Coronavirus Prevention from BRAC HNPP program? | 1=Yes  0=No | If response is ‘yes’ go to timing_train, train_dura, times_train & topic_train |
| timing_train | When did you receive the first training or orientation? | 1=November 2020  2=December 2020  3=January 2021  4=February 2021  5=March 2021 |  |
| train_dura | Duration of the first training or orientation? | 1= 1 hour  2= 2 hours  3= Half day  4= 1 day  5= 2 days |  |
| times_train | Till date, how many training or orientation did you receive? | --------------- times |  |
|  | Did you get any refresher trainings? | 1=Yes  0=No |  |
|  | If yes, how many? | --------------- times |  |
| topic_train | What are the topics covered during the training or orientation?  [multiple responses] | 1= Idea about COVID-19  2= How COVID-19 transmit/spread  3= Sign and symptoms of COVID-19  4=Hand washing and other hygiene practices  5= Use of masks  6= Social and safe physical distancing  7= Where to get COVID-19 test services  8= Other supports and services facilities  9= Identifying presumptive COVID-19 cases  10= Making referral linkage with CST for test and other services  11= Arrange isolation/quarantine of COVID-19 positive cases or their close contacts at their home  12= Arranging cost-effective hand washing stations at household and community  13= How to measure temperature  14= How to measure oxygen saturation  15=How to categorize PII, SPSC etc.  16=COVID 19 Vaccination  96= Others (specify………………..) |  |
|  | Did you know about any of these topics before getting the training? | 1= Yes  0= No |  |
|  | If yes, which topics did you know about from before? (multiple response) | 1= Idea about COVID-19  2= How COVID-19 transmit/spread  3= Sign and symptoms of COVID-19  4=Hand washing and other hygiene practices  5= Use of masks  6= Social and safe physical distancing  7= Where to get COVID-19 test services  8= Other supports and services facilities  9= Identifying presumptive COVID-19 cases  10= Making referral linkage with CST for test and other services  11= Arrange isolation/quarantine of COVID-19 positive cases or their close contacts at their home  12= Arranging cost-effective hand washing stations at household and community  13= How to measure temperature  14= How to measure oxygen saturation  15=How to categorize PII,SPSC etc.  16=COVID 19 Vaccination  96= Others (specify………………..) |  |
| bcc_mat1 | Have you received any BCC material from the project? [Please explain what does it mean by BCC material] | 1= Yes  0= No |  |
| bcc_mat2 | So far, what are the BCC material you received from this project?  [Please explain what does it mean by BCC material]  [Multiple response possible] | 1= Leaflets  2= Stickers  3= Module/guideline on the project  4= Poster  5= Booklet  96= Others (specify……………….) |  |
| bcc_mat3 | What Have you done with the bcc materials that you received? [Multiple response possible] | 1= Distribute in the neighbouring household  2= Distribute in the mosque  3= Distribute in the Bazar  4= Kept at my home  5= kept for my personal use  96= Others |  |
| comm_mat | What is the source of information that you have on COVID 19? | 1= Mobile phone (phone-call or message)  2= Newspaper/ Poster/ Banner/ Billboard  3= TV/ Radio  4= Facebook/ YouTube/ Instagram  5= Miking  6= Family/ Relatives  7= Friends  8=Neighbours  9= Colleagues  10= Govt. CHW from CC (CHCP/ HA/ FWA)  11= Govt. CHW from FWC (i.e., MO/ SACMO/ FWV/ Pharmacist)  12=Govt. CHW from other facilities  13= BRAC CHW (i.e., SS/ SK/ PK)  14=Other NGO CHWs  15=Village-doctor/ Drug seller/ Drug store owner  16=School teacher  17= Religious leader  18=Member/ Chairman of local union parishad  96=Others (specify)  97=Don’t know |  |
| comm_mat_eff | (If 6, 7, 8, or 9; for each of these four options ask separately for that person being referenced), ask whether that person belongs to this list | 1= Govt. CHW from CC (CHCP/ HA/ FWA)  2= Govt. CHW from FWC (i.e., MO/ 3=SACMO/ FWV/ Pharmacist)  4=Govt. CHW from other facilities  5=BRAC CHW (i.e., SS/ SK/ PK)  96=Other NGO CHWs  6=None of them |  |
|  | As per your experiences, which one is the most useful/influencing or effective? | 1= Mobile phone (phone-call or message)  2= Newspaper/ Poster/ Banner/ Billboard  3= TV/ Radio  4= Facebook/ YouTube/ Instagram  5= Miking  6= Family/ Relatives  7= Friends  8=Neighbours  9= Colleagues  10= Govt. CHW from CC (CHCP/ HA/ FWA)  11= Govt. CHW from FWC (i.e., MO/ SACMO/ FWV/ Pharmacist)  12=Govt. CHW from other facilities  13= BRAC CHW (i.e., SS/ SK/ PK)  14=Other NGO CHWs  15=Village-doctor/ Drug seller/ Drug store owner  16=School teacher  17= Religious leader  18=Member/ Chairman of local union parishad  96=Others (specify)  97=Don’t know |  |
|  | (If 6, 7, 8, or 9; for each of these four options ask separately for that person being referenced), ask whether that person belongs to this list | 1= Govt. CHW from CC (CHCP/ HA/ FWA)  2= Govt. CHW from FWC (i.e., MO/ 3=SACMO/ FWV/ Pharmacist)  4=Govt. CHW from other facilities  5=BRAC CHW (i.e., SS/ SK/ PK)  96=Other NGO CHWs  6=None of them |  |

**Part 6: Roles and responsibilities as a CST member: early Identification, management, & support component**

| **Sl. No.** | **Question** | **Codes/answers** | **Instruction** |
| --- | --- | --- | --- |
| cst_mem | Community support team (CST) is composed of?  [Multiple response possible] | 1= Shasthya Kormi (SK)  2= Health Assistant (HA)  3= Both  96= Others (specify…………..)  97 = Don’t know |  |
| task_cst | What are the activities of members of CST?  [Multiple response possible] | 1= Asses own health and take proper safety precautions  2= Identify Potential Infected Individuals/ Screened Positive Suspected Case  3= Connect individuals with symptoms to telemedicine  4= When necessary refer to nearest COVID testing/treatment facility  5= Ensure Home quarantine  6= Ensure Home quarantine of ultra-poor SPSC and ensure financial help for them  7= Enter patient data into tablets  8= Give health education/advice to PIIs and their family members  9= Keep contact with CCPC members  10= Identify people eligible for COVID 19 vaccinations  11= Do the online registration for people eligible for COVID 19 vaccinations  12= Raise awareness on 3 key protective measures during HH visits  13= Ensure maintenance of handwashing station at CC  96= Others (specify…………)  97 = Don’t know |  |
|  | How prepared do you feel to carry out the abovementioned tasks? | 1= Well prepared/ well trained  2= Averagely prepared  3= Not well prepared/ inadequately trained  96= others  97= don’t know |  |
|  | How often do you assess your own health? | 1= every day  2= every alternate day  3= 2 times a week  4= less than 2 times a week  5= never  97 = Don’t know |  |
|  | What are the ways you asses your own health? (multiple) | 1= take own temperature  2= check symptoms of cough  3= check symptoms of lack of smell  4= check symptoms of diarrhea  5= check symptoms of sore throat  6= check symptoms of lack of taste  96= Others (specify……………..)  97 = Don’t know |  |
|  | What do you do if you have any symptoms? (multiple) | 1= Tell the area manager  2= Take leave from work until recovered  96= Others (specify……………..)  97 = Don’t know |  |
|  | How do you maintain your own safety while providing services? (multiple) | 1= Wear mask  2= wear gloves  3= wear goggles  4= keep a hand sanitizer  5= wear face shield  6= maintain 3 feet distance  7= tell PIIs/SPSCs/patient to wear mask before speaking  8= Hold the thermometer 3-7cm away from patient’s forehead  9= Use hand sanitizer after every HH visit  96= Others (specify……………..)  97 = Don’t know |  |
|  | How do you maintain your own safety after coming back home from work? (multiple) | 1= wash clothes with soap and dry under sun after coming back home  2= take a shower after coming back home  3= don’t touch family members before disinfecting self  96= Others (specify……………..)  97 = Don’t know |  |
|  | What are the ways in which presumptive Covid cases are located? (multiple response) | 1= If a person calls the government hotline  2= from community people  3= during HH visits  4= family member of an identified PII  5= someone who came into contact with a COVID 19 patient  6= Someone who reported COVID 19 symptoms  96= others  97= don’t know |  |
| pvf_crit | Based on what criteria are presumptive Covid cases categorized? | 1= Symptoms, history and temperature  2= symptoms only  3= Temperature only  4= History only  96= others  97= don’t know |  |
| pvf_cat | How many categories are presumptive Covid cases divided in? | 1= 1  2= 2  3= 3 or more |  |
| pvf_cat_explain | What are the categories of presumptive Covid cases?  (multiple response) | 1= Screened Positive Suspected Case (SPSC)/Screened Cases (SCs)  2= Potential Infected Individuals (PIIs)  96= Others  97= don’t know |  |
| under_obspvf | Who are called PIIs? (multiple) | 1= Those who have a fever of 99F or below  2= Those who don’t have any of the symptoms related to breathing, such as, cough, sore throat, loss of smell, difficulty breathing.  96 = others  97= don’t know |  |
| vvf_cat | Who are called SPSCs/SCs? (multiple response) | 1= Those who have a fever of 100 F or more  2= Those who have any of the symptoms related to breathing, such as, cough, sore throat, loss of smell, difficulty breathing.  3= Those have tested positive for COVID-19  4= Those who have fever below 100 F but had paracetamol  96 = others  97= don’t know |  |
| Cst_resp_pvf | In case of SPSCs, what are the responsibilities of CST members? (multiple) | 1= Visit the patient’s house within 48 hours of knowing about them  2= Examine the SPSC and fill up their information into the mobile app  3= Communicate with doctors over phone and take the necessary measures  4= Give counselling to SPSC and their family members  5= Refer to the nearest COVID hospital in case of severe breathing difficulty  6= Wash hand/sanitize after every visit  7= Ensure safety of at-risk individuals  8= Follow up after 3 days by phone  9= Follow up after 7 days by phone  96 = others  97= don’t know |  |
|  | What are the advices/counselling to be given to SPSCs? (multiple) | 1= Begin home isolation of SPSC immediately  2= maintain 3 feet distance with other family members  3= Wear mask at all times  4= wash cloth mask with soap and dry under the sun after use  5=Cough/sneeze into tissue/cloth/elbow  6= discard tissue in closed bin and wash cloth with soap and water  7= Stay clean and Wash hands for 20 seconds with soap and water frequently  8= Use separate clothes, bed, utensils  9= Talk with other family members  10= Eat nutritious food including vitamin C  11= Call the hotline or the CST member in case of any problems  96 = others  97= don’t know |  |
|  | What are the advices/counselling to be given to SPSC’s family members?? (multiple) | 1= All family members should stay home for 14 days  2= maintain 3 feet distance between each other  3= Other people must bring food/ medicines from outside, or only 1 person can be assigned to it  4= No visitors should be allowed  5= Wash hands for 20 seconds with soap and water frequently  6= Cough/sneeze into tissue/cloth/elbow  7= discard tissue in closed bin and wash cloth with soap and water  8= Disinfect frequently touched surfaces (doorknob, mobile)  9= Disinfect living room and bathroom floors  10= Stay clean and wash used clothes and furniture with soap and water  11= If there are any elderly (60+), pregnant, or chronically ill individuals in the family, then relocate them for 14 days  96 = others  97= don’t know |  |
| cst_resp_vvf | In case of PII, what are the responsibilities of CST members?  (multiple) | 1= Explain to PII that they are not in the SPSC category  2= Tell them to stay at home and not go outside or invite anyone home unnecessarily  3= Must wear mask if they go outside  4= Maintain 3 feet distance and avoid crowds  5= tell them to wash hands for 20 seconds with soap and water frequently  6= Show them proper hand washing method  7= Cough/sneeze into tissue/cloth/elbow  8= discard tissue in closed bin and wash cloth with soap and water  9= If any family member gets fever or cough, call the hotline or CST member immediately  96 = others  97= don’t know |  |
| refer | After initiation of this project, how many patients with severe/critical symptoms had been referred to COVID 19 testing/treatment facility by you? | …………………patients  97 = Don’t know |  |
| refer2 | Do you provide them any referral slip? | 1= Yes  0= No |  |
| refer3 | If no, why not? | 1= There was no referral slip system in place  2= I was not asked to  3= I did not receive any referral slip from the BRAC office  4= No referral slips are needed at the referral facility  96= Other |  |
| refer4 | Do you also have to accompany the patients to the referral facility? | 1= Yes  0= No |  |
| refer5 | Do you follow up on the patients whom you have referred? | 1= Yes, always  2= Yes, most of the time  3= Yes, sometimes  4= No |  |
| refer6 | If no, why not? | 1= I do not have time to do so  2= I was not asked to do so  96= Other |  |
| refer7 | If yes (1,2,3), how frequently do you follow up on the referred individuals? | 1= After 3 days of referral  2= After 7 days of referral  3=Whenever I get some time to do so  4= Whenever I am asked to do so  96= Others |  |
| financial_sup | After initiation of this project, how many poor/ultra-poor COVID-19 patients got financial support for quarantine from BRAC by you? | …………………patients  97 = Don’t know |  |
| thermo_num | How many functional infrared thermometers do you have? | …………………many  97 = Don’t know |  |
|  | How many functional oximeters do you have? | …………………many  97 = Don’t know |  |
| tab_num | How many functional tabs do you have? | …………………many  97 = Don’t know |  |

**Part 7: Roles and responsibilities as a CST member: vaccination procedure**

| **Sl. No.** | **Question** | **Codes/answers** | **Instruction** |
| --- | --- | --- | --- |
|  | Did you receive any training on COVID 19 vaccination? | 1= yes  0= no |  |
|  | What were the topics covered in the training? | 1= Vaccine related communication  2= identifying eligible beneficiaries  3= online registration process  96 = others (--------)  97= don’t know |  |
|  | Who are eligible for COVID 19 vaccine? | 1= people over 40 years age  2= frontline health workers  3= people with special occupation (law enforcement, media, all government employees, bank officer, teacher, etc)  96= Others (specify……………..)  97 = Don’t know |  |
|  | How do you locate eligible beneficiaries of COVID 19 vaccine? | 1= during HH visit  2= through SS  3= through community members  96= Others (specify……………..)  97 = Don’t know |  |
|  | What are the information needed for online registration for COVID19 vaccine? | 1= NID  2= mobile number  96= Others (specify……………..)  97 = Don’t know |  |
|  | How many people do you register for vaccination per day? | ---------------- / day |  |
|  | What are the challenges/difficulties you face while doing online registration for beneficiaries? | 1=Slow internet access/ no network  2=Could not submit the form at first try  3=Beneficiary did not have the required documents (NID)  4= Beneficiary did not have a mobile number  5= I did not face any difficulties  96= Others (specify……………..)  97 = Don’t know |  |

**Part 8: Knowledge, attitude and practice on Vaccination**

| **Sl. No.** | **Question** | **Code** | **Options** |
| --- | --- | --- | --- |
| **Knowledge** | | | |
| qg_3 | Have you heard anything about a vaccine against the novel coronavirus? | 0 | No |
|  |  | 1 | Yes |
| qg_4 | (If Yes) What is the source of information that you have on vaccine? |  | 1= Mobile phone (phone-call or message)  2= Newspaper/ Poster/ Banner/ Billboard  3= TV/ Radio  4= Facebook/ YouTube/ Instagram  5= Miking  6= Family/ Relatives  7= Friends  8=Neighbours  9= Colleagues  10= Govt. CHW from CC (CHCP/ HA/ FWA)  11= Govt. CHW from FWC (i.e., MO/ SACMO/ FWV/ Pharmacist)  12=Govt. CHW from other facilities  13= BRAC CHW (i.e., SS/ SK/ PK)  14=Other NGO CHWs  15=Village-doctor/ Drug seller/ Drug store owner  16=School teacher  17= Religious leader  18=Member/ Chairman of local union parishad  96=Others (specify)  97=Don’t know |
| qg_5 | (If 6, 7, 8, or 9; for each of these four options ask separately for that person being referenced), ask whether that person belongs to this list |  | 1= Govt. CHW from CC (CHCP/ HA/ FWA)  2= Govt. CHW from FWC (i.e., MO/ 3=SACMO/ FWV/ Pharmacist)  4=Govt. CHW from other facilities  5=BRAC CHW (i.e., SS/ SK/ PK)  6=Other NGO CHWs  7=None of them |
| Qna_1 | If yes, who should get the vaccine? |  | 1= Everyone should get vaccinated  2=People over the age of 40 should get vaccinated  3=Nobody should get vaccinated  96=Others  97= Don’t know |
| qN_1 | How many BRAC COVID Vaccine assistance booths are there in this union? |  | ………… |
| qN_2 | Where can people get the vaccine from? |  | 1=CC  2=FWC  3=District hospital  4=Medical college hospital  5=Specialized hospital  6=Other public hospital/ clinic  7=Private hospital/ clinic  96=Others (specify)  97=Don’t know |
| Qna_2 | Is there any fee for getting vaccinated? (only for taking vaccine) |  | 0=No  1=Yes |
| Qna_3 | If yes, how much? |  | ---------------- |
| qN_6 | Do you know how to register for the vaccine? |  | 0=No  1=Yes |
| Qna_4 | What are the ways can you register for getting the vaccine? |  | 1=Online registration through govt. website/ app  2=In-person visit to a registration booth  3=Volunteers visit the households to register  96=Others (specify)  97=Don’t know |
| Qna_5 | Does the vaccine have any side effect? |  | 0=No  1=Yes  2=Not sure |
| qN_5 | (If yes) What are the possible side effects of the vaccine? |  | 1=Muscle pain/ body ache  2=Pain at the injection site  3=Swelling at the injection site  4=Redness at the injection site  5=Weakness/ fatigue  6=Nausea  7=Fever  8=Chills  9=Headache  96=Others (specify)  97=Don’t know |
|  | **Attitude** |  |  |
| Qna_6 | Are you willing to get vaccinated? |  | 0=No  1=Yes  3=Refuse to respond |
| Qna_7 | (If yes) Why are you willing to take it? |  | 1=To prevent COVID-19 or to prevent the novel coronavirus transmission  2=Because everyone else is having it  3=Because I have been asked to do so  4=Because It is being administered free-of-cost  96=Others (specify)  99= Refuse to respond |
| Qna_8 | (If no) Why are you not willing to take it? |  | 1=I do not think the vaccine is necessary  2=I do not know what they are giving me  3=Afraid about its side effects  4=Afraid of injections  5=Not permitted in my religion  6=The entire vaccine thing is a farce  7=There is no such thing as COVID-19 or novel coronavirus here  96=Others (specify)  99=Refuse to respond |
| Qna_9 | Are your family members willing to get vaccinated? |  | 0=No  1=Yes |
| qg_7 | If Yes: Who in your family would you prefer taking it first? |  | 1=Entire family  2=Children only  3=Pregnant  4=Elderly  5=Males of the household  6=Females of the household  7=Respondent themselves  96=Others (Specify) |
| qN_4 | Do you think that the vaccine is safe? |  | 0=No  1=Yes  3=Not sure |
|  | **Practice** |  |  |
| Qna_10 | Did you register for vaccine? |  | 0=No  1=Yes |
| Qna_11 | (If no) Why did you not register? |  | 1=I don’t want to take the vaccine  2=I don’t know how or where to register  3=I do not have internet access.  4=I don’t know the web link  5=Could not open the web link  6=Find it difficult to fill the form  7=I do not find anyone to fill out the form for me  8=I was busy  9=I do not match any of the eligibility criteria  96=Others (specify) |
| Qna_12 | If yes, how did you do it? |  | 1=Online  2=Went to a BRAC COVID Vaccination Assistance booth  3=A volunteer visited my house and did it for me  Others (specify) |
| Qna_13 | If yes, did you download a vaccine card? |  | 0=No  1=Yes |
| Qna_14 | If yes, did you print it out? |  | 0=No  1=Yes |
| Qna_15 | If yes, do you have the vaccination card? (Check to see it) |  | 0=No  1=Yes, observed  2=Yes, not observed |
| qN_10 | If yes, what are the difficulties you faced to register? |  | 1=The user interface was difficult to understand  2=Could not submit the form at first try  3=I had to manage computer/ internet access  4=I had to find someone who can fill out the form for me  5=I didn’t face any difficulties  96=Others (specify) |
| Qna_16 | Have you got the first dose of vaccine? |  | 0=No  1=Yes |
| qN_7 | Has any other member of your household register for the vaccine? |  | 0=No  1=Yes |
| qN_13 | Has any other member of your household received the first dose of vaccine? |  | 0=No  1=Yes |
| qN_14 | Did you/ any of your household members face any difficulty to get the vaccine? |  | 0=No  1=Yes |
| qN_15 | (If yes) What are the difficulties did you/ your household members face while receiving it? |  | 1=Had to travel a long distance  2=Transport cost was too high  3=Had to wait in a queue for long  4=Got some of the side effects  5=Did not take the required documents (NID, Vaccination Card)  6=Could not show the SMS sent by govt.  96=Others (specify) |
| Qna_17 | How long did it take for you to reach the centre? |  | ------------minutes |
| Qna_18 | How long did you have to wait in the queue to get the shot? |  | ______minutes |
| Qna_19 | What would be your recommendations to improve the system further? (multiple) |  | 1=Assistance in registration  2=Assistance in travelling to the centre  3=The vaccine should be made available for everyone irrespective of the eligibility  4=More vaccines should be procured  5=Should be administered to all free-of-cost  6=No recommendation  96=Others (specify) |
| qN_16 | (For those who got the first dose) Would you be willing to get the second dose? |  | 0=No  1=Yes  3=Not sure |
| Qna_20 | (If no) Why not? |  | 1=Had a bad experience while receiving the first dose  2=Got some of the side effects  3=COVID-19 or the novel coronavirus transmission is under control now  4=Do not need another shot  96=Others (specify) |

**-------------------Thank you very much for your valuable time ☺--------------------**

**IR components: KII protocol and guideline**

Research on the “Community Based Comprehensive COVID 19 Response Project”

BRAC James P Grant School of Public Health, BRAC University, Dhaka

**Part 1: Background information**

| **Respondents’ information** | | **Interview information** |
| --- | --- | --- |
| Respondent ID: | | Name of the interviewer: |
| Name: | | Name of note taker: |
| Sex: | Age: | Interview starting time: |
| Educational attainment: | | Interview ending time: |
| Current designation: | | Date of interview: |
| Total years of work experience: | |  |
| **Place of interview:**  Village: Union: | | |

**Part 2: KII questions**

**Opening topic (10 minutes)**

**A. Overall Health-related**

- What is the main health related problems in your community?
- How can those problems be solved/ addressed?
- How can community members be engaged in solving those problems?
- Is there any way you could contribute to address these problems and how?

**B. About the COVID-19 project**

- What is your understanding regarding goal/objective of this COVID-19 project?
- From when are you involved in this project? What is your role in implementing this COVID-19 project?
- Who do you report to regarding your work on this project (How/to whom/who reports to you)?
- Did you receive any orientation (CCPC) or training (Formal provider) before starting work in this project? If yes, tell us about the duration, date, number of sessions and topic covered in the training.

**Topic questions (30 minutes)**

**A: Perception on implementation of comprehensive COVID 19 response activities**

- What activities are being delivered by BRAC to community through this project? *[Prompts: 3 key preventive practices, signs/symptoms, quarantine, isolation, referral and care]*
- What is your perception about the effectiveness and community acceptability of the intervention? *[Prompts: awareness raising in the community, early identification of case and referral, provision of required medicine, and behavior surveillance among four specific population groups]*
- Effectiveness of community forums. *[Prompts: CCPC and CST].*
- Do you have the confidence/belief that this project is going to improve the COVID-19 situation in this community? If yes, how? If no, why not? *[Instruction: please explain briefly with examples]*

**B: Bottlenecks in implementing comprehensive COVID 19 response activities**

- **Are you or your team facing any challenges/barriers/constraints during the implementation of this project? If yes, what are those? Can you please share any specific story/incident regarding the challenges faced?** *[Instructions: Ask separately for each of the following topic]:*
  - During the formation of different groups (CCPC, CST etc.)
  - While coordinating the different stakeholders of the project (as this project is implemented by members from different BRAC programs and also involves community members and local health care providers)?
  - What challenges are faced regarding BCC materials?
    - While distributing materials and disseminating information (such as, handwashing, mask-wearing, and physical distancing)
    - While procuring/collecting materials (like masks, soaps, etc.)
  - Any constraints from demand perspectives (from the community people)
  - Regarding the acceptability of the interventions by community people
  - Regarding any point of the whole process of delivering the services (CCPC: while raising awareness and distributing masks and soap; quarantine, isolation, follow-up, referral, and care etc.)
  - Regarding arranging cost-effective hand washing stations
  - Regarding the supply chain: What were the challenges faced while arranging for the required medicines, pulse oximeters, thermometers, network, and other support? Give details.

**C: Facilitators in implementing comprehensive COVID 19 response activities**

- What are the best parts/ positive aspects (strategies, plans, actions etc.) of implementation of this project? Can you please describe any relevant situation/incident/story? Give details.

**D: Moving forward in implementing comprehensive COVID 19 response activities**

- How do you think we can overcome the implementation barriers, challenges (for better future implementation) of this project?
- How did you solve/cope with the challenges you mentioned? *[Prompt: refer to the challenges mentioned in previous sections]*
- What is your idea/suggestion/recommendation for improving the implementation of this project?

**Closing questions (5 minutes)**

*Thank you again for sharing valuable information. We really appreciate your participation. Now, we would like to close our discussion. Would you please describe …*

- Is there anything else we have not discussed that you would like to say about implementing the comprehensive COVID 19 response activities in your area? If yes, please share.

**-------------------Thank you very much for your valuable time ☺--------------------**

**IR components: Handwashing Station Observation Checklist**

Research on the “Comprehensive COVID-19 Response through Community Mobilization

and Strengthening Community Clinics”

BRAC James P Grant School of Public Health, BRAC University, Dhaka

**Observation period: 30 minutes (for each place)**

**Part 1: Identification and interview related information**

| **Sl. No.** | **Question** | **Codes/answers** | **Instruction** |
| --- | --- | --- | --- |
| surveyor | Name of the Surveyor |  |  |
| date | Date of the survey |  |  |
| district | District Name | 1=Bogura  2=Narayanganj  3=Bhola |  |
| upazila | Name of the Upazilla |  |  |
| union | Name of the Union |  |  |
| village | Name of the Village |  |  |
| ccpc | Name of the CC | __________________ |  |
| reid | Questionnaire ID: |  |  |

**Part 2: Presence of Handwashing Station and utilization of the facility**

| **Sl. No.** | **Question** | **Code** | **Options** | |
| --- | --- | --- | --- | --- |
| 1 | Location of handwashing station |  | 1 = Poor/ ultra-poor Household  2 = Community clinic (CC) | |
| 2 | How many water taps were there in the handwashing station? |  |  | |
| 3 | Is there water in the handwashing station?  [Turn the tap on and note if running water is present  OR Look for any container where water is present] |  | 1 = Yes  0 = No | |
|  | What is the source of the water of the handwashing station? |  | 1= supply water  2= tube well  3= stored water (in a container)  96= others | |
| 4 | Is there any soap or detergent or locally used cleansing agent? |  | 1 = Handwashing soap bar  2 = Liquid soap  3 = Laundry detergent/soap  4 = Ash  5 = Mud/Sand  6= No cleansing agent  96 = Others (specify) | |
|  | **Utilization of hand washing station** | | | |
| 5 | During the observation period, how many people went to the hand washing station? |  | ………………………person | |
| 6 | How many of them washed their hands in the hand washing station? |  | ………………………. person | |
|  | **How many of them followed the steps of proper hand washing? [among those who had washed their hand in hand washing station]** | | | |
|  |  |  | **No. of People Observed within 30 minutes** | **No. of People who Followed within 30 minutes** |
| a | Wet hands with water |  |  |  |
| b | Apply enough soap to cover all hand surfaces |  |  |  |
| c | Rub hands palm to palm |  |  |  |
| d | Right palm over left dorsum with interlaced fingers and vice versa |  |  |  |
| e | Palm to palm with fingers interlaced |  |  |  |
| f | Backs of fingers to opposing palms  with fingers interlocked |  |  |  |
| g | Rotational rubbing of left thumb clasped in right palm and vice versa |  |  |  |
| h | Rotational rubbing, backwards and forwards with clasped fingers of right hand in left palm and vice versa |  |  |  |
| i | Rinsing both hands with water |  |  |  |
| J | Scrub hands for at least 20 seconds |  |  |  |
