# Supplementary material for: Implementation barriers and facilitators to a COVID-19 intervention in Bangladesh: The benefits of engaging the community for the delivery of the programme
Source: BMC Health Serv Res. 2022 Dec 28;22:1590. doi: 10.1186/s12913-022-08939-7 (PMC9795148; doi:10.1186/s12913-022-08939-7)
Supplement: Supplementary file 1 [file 12913_2022_8939_MOESM1_ESM.docx]

**Supplementary file** 

*Supplementary Table 1: List of project activities to create community resilience* 

| **Types of resilience capacity** | **Project activities** |
| --- | --- |
| **Adaptive resilience:** The ability of community to adjust with COVID-19 pandemic situation. | Involving community people as implementing agent through the formation of CCPC Capacity building (orientation/ refreshers) of the CCPC and CST members Awareness rising at community level about preventive measures, sign symptoms, transmission through household visits and monthly meeting |
| **Absorptive resilience:** The ability to absorb and effectively cope with COVID-19; the capacity to manage and recover from adverse conditions, using available skills, assets and resources. | Masks, soaps, and handwashing stations distribution among the poor Ensuring home quarantine and home isolation supports for the poor (food, medicine, transportation support) Linking to referrals (Upazila health complex and other tertiary level facilities) |
| **Anticipatory resilience:** The ability to reduce risks by means of proactive action to minimize vulnerability. | Practice of preventive measures of COVID-19 by implementers as well as community people Early identifying of active case of the COVID-19 |
| **Transformative resilience:** The ability of systems to transform their structures and means of operating to better address change and uncertainty due to COVID-19 and to develop (new) systems that are more suited to new conditions. | Collaboration with local government health system Telemedicine services |

Supplementary Table 2: Sampling strategy and sample size for the quantitative survey   

| **Selection tiers** | **Sampling strategy** | **Highland** | **Plain** | **Coastal** |
| --- | --- | --- | --- | --- |
| **Districts (3)** – one from each type of geographical region | Purposive | Bogura | Narayanganj | Bhola |
| **Sub-districts (3)** – three from each district | Simple random sampling | 3 (Dupchachia, Gabtoli, Shobganj) | 3 (Araihajar, Rupganj, Sonargaon) | 3 (Monpura, Borhanuddin, Tajmuddin) |
| **Community Corona Protection Committee/CCPC (60)** | Simple random sampling | 20 | 20 | 20 |
| **Community Support Team/CST (60)** | Simple random sampling | 20 | 20 | 20 |

Supplementary Table 3: Qualitative study participants by study areas

| **Respondents (implementing agents) [N=54]** | **Bogura** | **Narayanganj** | **Bhola** |
| --- | --- | --- | --- |
| BRAC implementers at field level |  |  |  |
| Area Manager (AM) | 1 | 1 | 2 |
| Telemedicine doctor | 1 | 1 | 1 |
| Field Organizer (FO) | 4 | 4 | 3 |
| *Shasthya Kormi* (SK) | 3 | 4 | 5 |
| *Shasthya Shebika* (SS) | 3 | 3 | 5 |
| Government providers at field level |  |  |  |
| Health Assistant (HA) /Family Welfare Assistant (FWA) | 3 | 3 | 3 |
| Community Health Care Provider (CHCP) | 1 | 1 | 0 |
| BRAC Divisional Manager/DM (Central/Head Office) | 1 | 0 | 1 |
| **Total** | **17** | **17** | **20** |

Supplementary Table 4**:** Topic discussed during the training of the CCPC and CST members (only among those who received training)

| ***Indicators*** | **CCPC members** | **CST members** **(n=68)** |  |
| --- | --- | --- | --- |
| **Topics discussed in the training reported by the CCPC members (Self-reported)** | **(n=472)** | **SKs** **n=38** | **HAs** **n=30** |
| Signs and symptoms of COVID-19 | 354 (75.00) | 34 (89.47) | 26 (86.67) |
| COVID-19 preventive practices: Use of masks, hand washing, & social distancing | 197 (41.74) | 18 (47.37) | 12 (40.00) |
| General idea about COVID-19 and its transmission | 185 (31.19) | 27 (71.05) | 26 (86.67) |
| Ways to identify presumptive COVID-19 cases | 90 (18.33) | 8 (21.05) | 9 (30.00) |
| Available supports and services facility for COVID-19 treatment | 71 (14.46) | 6 (15.79) | 5 (16.67) |
| Testing center for COVID-19 | 67 (13.65) | 7 (18.42) | 5 (16.67) |
| Ways to arrange isolation/quarantine of positive COVID-19 cases and contacts at home | 66 (13.44) | 6 (15.79) | 2 (6.67) |
| Ways to set up cost-effective handwashing stations at HH level and in the community | 58 (11.81) | 7 (18.42) | 3 (10.00) |
| About vaccination of COVID-19 | 50 (10.59) | 6 (15.79) | 5 (16.67) |
| Making referral linkage between CST members and testing and treatment facility | 28 (5.70) | - | - |
| Creating referral link with different COVID-19 testing facility^†^ | - | 7 (18.42) | 4 (13.33) |
| Ways to measure temperature^†^ | - | 12 (31.58) | - |
| Way to measure oxygen saturation^† ^ | - | 7 (18.42) | - |
| Way to categorize PII^‡^, SPSC^§ ^etc.^ †^ | - | 3 (7.89) | - |

†= these activities were only applicable for CST members;  ‡= Potential Infected Individuals;  §= Screened Positive Suspected Case
